# Analysis of differentially expressed genes among human hair follicle–derived iPSCs, induced hepatocyte-like cells, and primary hepatocytes

**DOI:** 10.1186/s13287-018-0940-z

**Published:** 2018-08-09

**Authors:** Ziran Xu, Xia He, Xu Shi, Yuhan Xia, Xiaomei Liu, Haitao Wu, Pengdong Li, Hongyu Zhang, Weisi Yin, Xiubo Du, Lisha Li, Yulin Li

**Affiliations:** 1 The Key Laboratory of Pathobiology, Ministry of Education, Norman Bethune College of Medicine, Jilin University, Changchun, 130021 People’s Republic of China; 2 Central Laboratory, The First Hospital of Jilin University, Changchun, 130021 People’s Republic of China; 3 Beijing Hospital of Traditional Chinese Medicine, Beijing, 100069 People’s Republic of China; 4 College of Life Sciences and Oceanography, Shenzhen Key Laboratory of Microbial Genetic Engineering, Shenzhen University, Shenzhen, People’s Republic of China

**Keywords:** Induced pluripotent stem cells, Stem cell differentiation, Hepatocyte-like cells, Differentially expressed genes, Microarray analysis

## Abstract

**Background:**

Differentiation of human induced pluripotent stem cells (hiPSCs) into hepatocytes has important clinical significance in providing a new stem cell source for cell therapy of terminal liver disease. The differential gene expression analysis of hiPSCs, induced hepatocyte-like cells (HLCs), and primary human hepatocytes (PHHs) provides valuable information for optimization of an induction scheme and exploration of differentiation mechanisms.

**Methods:**

Human hair follicle-derived iPSCs (hHF-iPSCs) were induced in vitro by mimicking the environment of a developing liver for 19 days. Expression of specific proteins was determined by immunofluorescence staining; the function of HLCs in storage and metabolism was identified by detecting periodic acid–Schiff, indocyanine green, and low-density lipoprotein. Based on the transcriptomics data, the differential gene expression profiles of hHF-iPSCs, HLCs, and PHHs were analyzed by Gene Ontology, Kyoto Encyclopedia of Genes and Genomes pathway, FunRich, and network analysis methods.

**Results:**

HLCs were able to express albumin (ALB), alpha-fetoprotein, CYP3A4, and CYP7A1, and exhibited matured liver cell functions such as glycogen synthesis and storage. Complement and coagulation cascades and metabolic pathways ranked top in the downregulated list of HLCs/PHHs, while the cell cycle ranked top in the upregulated list of HLCs/PHHs. In the protein–protein interaction network, according to the degree rankings, *TOP2A*, *CDK1*, etc. were the important upregulated differentially expressed genes (DEGs), while *ALB*, *ACACB*, etc. were the major downregulated DEGs in HLCs/PHHs; the module analysis indicated that *CDCA8*, *AURKB*, and *AURKA* were the top upregulated DEGs in HLCs/PHHs.

**Conclusions:**

We presented the differences in gene expression among hHF-iPSCs, HLCs, and PHHs through transcriptome array data and provided new ideas for the optimization of induction.

**Electronic supplementary material:**

The online version of this article (10.1186/s13287-018-0940-z) contains supplementary material, which is available to authorized users.

## Background

Liver transplantation is used to treat many liver diseases; however, donor insufficiency is a major challenge that has greatly reduced the feasibility of liver transplantation. In order to overcome the limitations of obtaining human hepatocytes from donor livers, hepatocyte-like cells (HLCs) have been obtained from other cells [1, 2]. Human induced pluripotent stem cells (hiPSCs) have similar self-renewal and differentiation potential to embryonic stem (ES) cells, and do not involve ethical issues [3], and thus they provide a new and suitable seed cell source for liver transplantation. At present, HLCs with drug metabolism and liver regeneration have been successfully obtained from hiPSCs in vitro [1], and hiPSC-derived HLCs are usually induced gradually from hiPSCs using a method similar to ES cells in vivo [4], by adding a suitable amount of bone morphogenetic protein (BMP), hepatocyte growth factor (HGF), and fibroblast growth factor (FGF).

Human hair follicles are a noninvasive, rich cell source. We obtained human hair follicle-derived iPSCs (hHF-iPSCs) possessing the potential for differentiating into HLCs through definitive endoderm (DE) induction via growth factors [5, 6], by means of the identification of protein expression levels, storage function, and the metabolism of glycogen, and determined the function of HLCs and the feasibility of differentiation. We believe that using this method for inducing the differentiation of hESCs into hepatocytes along with the accumulated experience in inducing differentiation of hHF-iPSCs, it would be possible to optimize the technical operation and explore the mechanism of hHF-iPSC differentiation into hepatocytes, which will provide a new way to solve the problem of cell source and lay the foundation for the wide application of iPSCs in the treatment of clinical liver disease.

In recent years, several omic studies have been conducted on hiPSCs and HLCs, but differences in cell sources, data, and analytical procedures can lead to different results [7–10]. To further optimize our induction protocol and reveal the molecular mechanism of stem cell-derived hepatocytes, Human Transcriptome Array 2.0 (HTA2.0) analysis of cells (hHF-iPSCs, HLCs, and primary human hepatocytes (PHHs)) was used for gene expression profiling of the three groups. A combination of transcriptomics analysis and genome-wide gene expression profiles of hHF-iPSCs to HLCs was investigated. In addition, the gene profile differences between HLCs and PHHs were obtained to construct differential expression networks, which were combined with network analysis methods to identify key molecules, such as hub nodes, in order to obtain group-level data to support new induction programs and signal transduction mechanisms.

## Methods

### hHF-iPSC culture

MEFs from CF-1 mice and hHF-iPSCs were cultured as described in a previous study [6]. The primary MEFs were cultured to passage 3 and treated with Mitomycin C (10 μg/ml) in order to obtain feeder cells suitable for iPSC growth. MEFs were cultured in MEF medium (DMEM supplemented with 10% FBS, 1% nonessential amino acids, 1 mM l -glutamine; all from Gibco, Invitrogen, USA). hHF-iPSCs were acquired by reprogramming human hair follicle-derived MSCs using a cocktail of lentiviruses carrying Yamanaka factors (*OCT4*, *SOX2*, *C-MYC*, and *KLF4*) [6] and cultured in a T25 culture flask coated with feeder cells in HES medium (80% DMEM/F12 supplemented with 20% KSR, 1% nonessential amino acids, 1 mM l-glutamine, 4 ng/ml human bFGF, and 0.1 mM β-mercaptoethanol; all from Invitrogen, USA). hHF-iPSCs were separated with 1 mg/ml collagenase type IV (Invitrogen, USA) for 30–60 min at 37 °C, at a ratio of 1:3 every 6–7 days. Conditioned medium was collected as described previously [11]. Y27632 (formulated with DMSO) was added to improve the efficiency of cell resuscitation.

### Hepatic induction of iPSCs in vitro

**Fig. 1:**
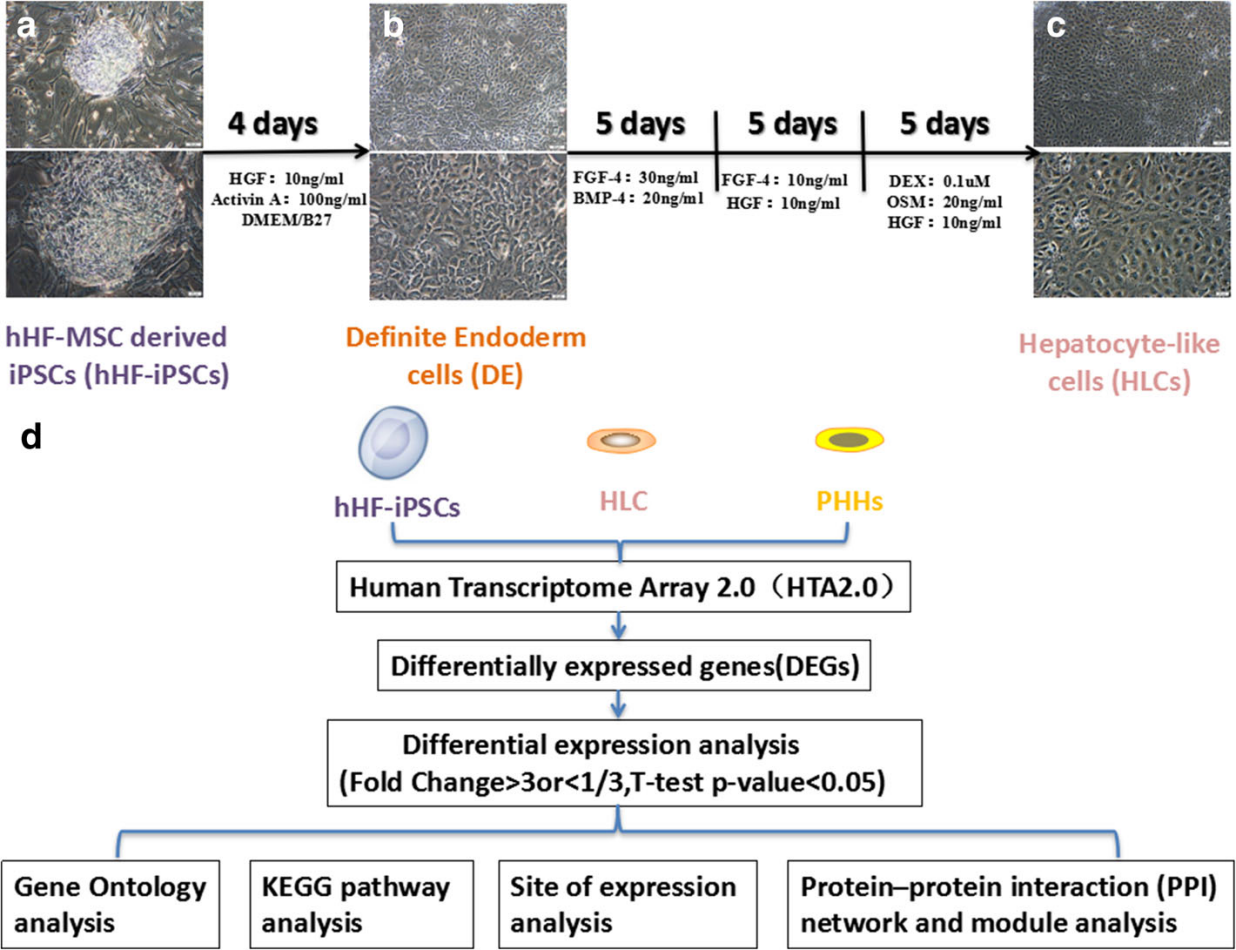
Differentiation of HLCs from hHF-iPSCs through DE. **a**–**c** Bright-field images; scale bars = 50 and 20 μm for top and bottom images, respectively. **d** Data analysis route used in this study. BMP bone morphogenetic protein, DEX dexamethasone, FGF fibroblast growth factor, HGF hepatocyte growth factor, hHF-iPSC human hair follicle-derived induced pluripotent stem cell, KEGG Kyoto Encyclopedia of Genes and Genomes, MSC mesenchymal stem cell, OSM oncostatin-M, PHH primary human hepatocyte

hHF-iPSCs were passaged on a fresh plate precoated with Matrigel evenly. Post adherence, the cells began to differentiate and the following day was considered to be day 1. With regard to the differentiation of ES cells or iPSCs into HLCs, we divided the induction process into four stages. Firstly, hHF-iPSCs were induced to DE in the first 4 days, and DMEM/F12 differentiation medium was selected and supplemented with 2% B27 (Invitrogen), 1% nonessential amino acids, 1 mM l-glutamine, and 100 ng/ml Activin A (ACT A; R&D Systems). Secondly, DE was differentiated into the hepatic specialization stage, which occurred from days 5 to 9. Differentiation medium was changed to HCM (Lonza) supplemented with 20 ng/ml BMP-4 (R&D Systems) and 30 ng/ml FGF-4 (Invitrogen). Thirdly, specialized cells were cultured in the culture medium containing 10 ng/ml HGF and 10 ng/ml FGF-4 for 5 days, and during this stage we could obtain liver progenitor cells. In the last stage, hepatic progenitor cells transformed into mature hepatocytes. To achieve this, DMEM/F12 medium was supplemented with 20 ng/ml oncostatin-M (OSM), 0.1 μM dexamethasone (DEX), and 10 ng/ml HGF during the last 5 days. Culture medium was replaced every 2 days (Fig. 1a).

### Acquisition of human hepatic tissue

Human hepatic tissue was acquired from postoperative samples of hemangioma patients. Fresh hepatic tissue was embedded in OTC, and then used for the preparations of frozen sections. The other portion of the fresh hepatic tissue was directly preserved in liquid nitrogen for microarray analysis.

### Immunofluorescence

To quantify the protein expression level of HLCs, hHF-iPSCs were used as the control group, and ALB, AFP, CYP3A4, and CYP7A1 were selected as hepatocyte marker proteins, which were identified by immunofluorescence staining. hHF-iPSCs, HLCs, and frozen sections of hepatic tissue were fixed with 4% paraformaldehyde (Biolegend) for 20 min and then permeabilized by 0.1% Triton-X in PBS for 10 min. Cells were then blocked with 5% BSA for 1 h and stained with primary antibodies (SOX17, ALB, AFP, CYP3A4, and CYP7A1) at 4 °C overnight. All of the primary antibodies diluted at a ratio of 1:100 were from Santa Cruz Biotechnology. The primary antibody which was used to detect the formation of DE was mouse anti-SOX17 antibody. Additionally, primary antibodies including mouse anti-ALB, rabbit anti-AFP, rabbit anti-CYP3A4, and mouse anti-CYP7A1 were selected to detect the corresponding genes. Cells were washed three times with PBS before incubation with secondary antibodies: Alexa Fluor 488-conjugated goat anti-mouse IgG (1:500; Invitrogen) and Alexa Fluor 594-conjugated goat anti-rabbit IgG (1:500; Invitrogen). Nuclei were stained with DAPI (US Everbright Inc.) at 25 °C for 2 min. Images were captured with an Olympus IX71 fluorescence microscope. We used ImageJ to quantify the percentage of positive SOX17, ALB, AFP, CYP3A4, and CYP7A1 populations from three pictures.

### Periodic acid–Schiff stain

To assess glycogen accumulation, differentiated cells were analyzed with a Periodic acid–Schiff (PAS) stain kit (Sigma-Aldrich). The cells were fixed with 4% paraformaldehyde and then oxidized in 1% periodic acid. After washing three times with ddH_2_O, the cells were incubated with Schiff’s reagent for 20 min. The cells were then treated three times with sulfite to achieve differentiation. Nuclei were counterstained with hematoxylin. Cells were visualized under a light microscope.

### Uptake of indocyanine green

The working solution was prepared by dissolving indocyanine green (IGG) (Sigma-Aldrich) in DMSO followed by the addition of this solution to differentiation medium at a final concentration of 1 mg/ml. After incubation for 1 h at 37 °C and 5% CO_2_, the medium with ICG was discarded and fresh medium was added to continuously culture the cells. Cells were examined and photographed by an inverted IX71 fluorescence microscope for 8 h.

### Uptake of low-density lipoprotein

Uptake of low-density lipoprotein (LDL) by HLCs was evaluated by the LDL Uptake Cell-Based Assay Kit (Cayman Chemical). After discarding the media, the cells were stained for 4 h in culture medium containing LDL-Dylight 550 at 37 °C and 5% CO_2_. Hoechst (diluted 1:10,000 in ddH_2_O) staining was used to counterstain cell nuclei at 25 °C for 2 min. An inverted fluorescent microscope was used to detect the degree of LDL uptake and photographic recording.

### Microarray data

In this experiment, the latest HTA2.0 from Affymetrix Company was used to analyze gene expression profiles of hHF-iPSCs, HLCs, and PHHs (from human hepatic tissue). This gene chip obtained more selective and splicing information than RNA-seq, and the information gained was in full agreement with the results of qRT-PCR verification. The design and annotation databases of the HTA2.0 chip were based on the most up-to-date information from the following databases: RefSeq, Ensembl, Rat Genome Database (RGD), MGC Mammalian Gene Collection, NONCODE, lncRNA db, and AceView. There were approximately 10 probes per exon and four probes per exon–exon splice site. This analysis could fully and accurately reflect the entire transcriptome expression data, including gene level, transcript level, and protein coding or noncoding RNA level.

### Gene Ontology and pathway enrichment analysis of differentially expressed genes

Gene Ontology (GO) is a major bioinformatics method for annotating genes and gene products identifying the characteristic biological properties of high-throughput genomic or transcriptome data, based on a rigorous biological background and adopting a unified database of structural annotation genes to standardize genes from molecular functions, biological processes, and cellular components, respectively [12, 13]. KEGG was used for pathway analysis of differentially expressed genes (DEGs) referring to the metabolic pathway, which is significantly changed under the known experimental conditions. Therefore, it is particularly important to study this underlying mechanism. We used DAVID (https://david.ncifcrf.gov/) to conduct a correlation analysis [14], to analyze the functional levels of DEGs, KEGG pathway, and GO enrichment, including GOTERM_MF_FAT, GOTERM_CC_FAT, and GOTERM_BP_FAT, which described the molecular function (MF), cellular component (CC), and biological process (BP) of the DEGs. In addition, test *P* < 0.05 was considered significant. All DEGs were uploaded to the online software DAVID to identify overrepresented GO categories and KEGG pathways.

### FunRich site of expression analysis of DEGs

FunRich is standalone software used primarily for functional enrichment and interaction network analysis of genes and proteins. Besides, the results of the analysis can be depicted graphically in the form of Venn diagrams, Bar, Column, Pie, and Doughnut charts. Currently, the FunRich tool is designed to handle a variety of gene/protein data sets irrespective of the organism. Additionally, users have more than 13,320 different background database options. One of the community-requested features is FunRich’s ability to update the back-end database in real time [15]. Users not only can search against the default background database but can also load a customized database against which functional enrichment analysis can be carried out. According to the site of expression analysis of the software, the upregulated and downregulated DEGs of HLCs hHF-iPSCs, HLCs/PHHs, and PHHs/hHF-iPSCs were analyzed, respectively. The top five sites of expression analysis were selected based on the percentage of DEGs, and pictures were plotted with abscissa –log_10_(*P* value). In addition, test *P* < 0.05 was considered significant.

### Integration of protein–protein interaction network and module analysis

The Search Tool for the Retrieval of Interacting Genes/Proteins (STRING) database (http://string-db.org) used for retrieving interactive genes is an online tool aimed to assess protein–protein interaction (PPI) information. STRING (version 9.0) covers 5214,234 proteins from 1133 organisms [16]. To estimate the interactions among DEGs, we mapped the DEGs to STRING, and  only experimentally validated interactions with a combined scores > 0.4 were selected as significant. Further, the PPI network was built and analyzed using Cytoscape [17]. The Molecular Complex Detection (MCODE) plug-in was used for screening PPI networks in Cytoscape [18]. The standard setting comprised MCODE score > 3 and quantity node > 30. After acquiring the subnetwork with the highest MCODE score, the color and size of its nodes were adjusted according to the MCODE score of DEGs, and the color and thickness of the connection were adjusted according to edge-betweenness. The final network diagram was obtained. In addition, test *P* < 0.05 was considered significant.

## Results

### Differentiation of hHF-iPSCs to HLCs through DE

**Fig. 2:**
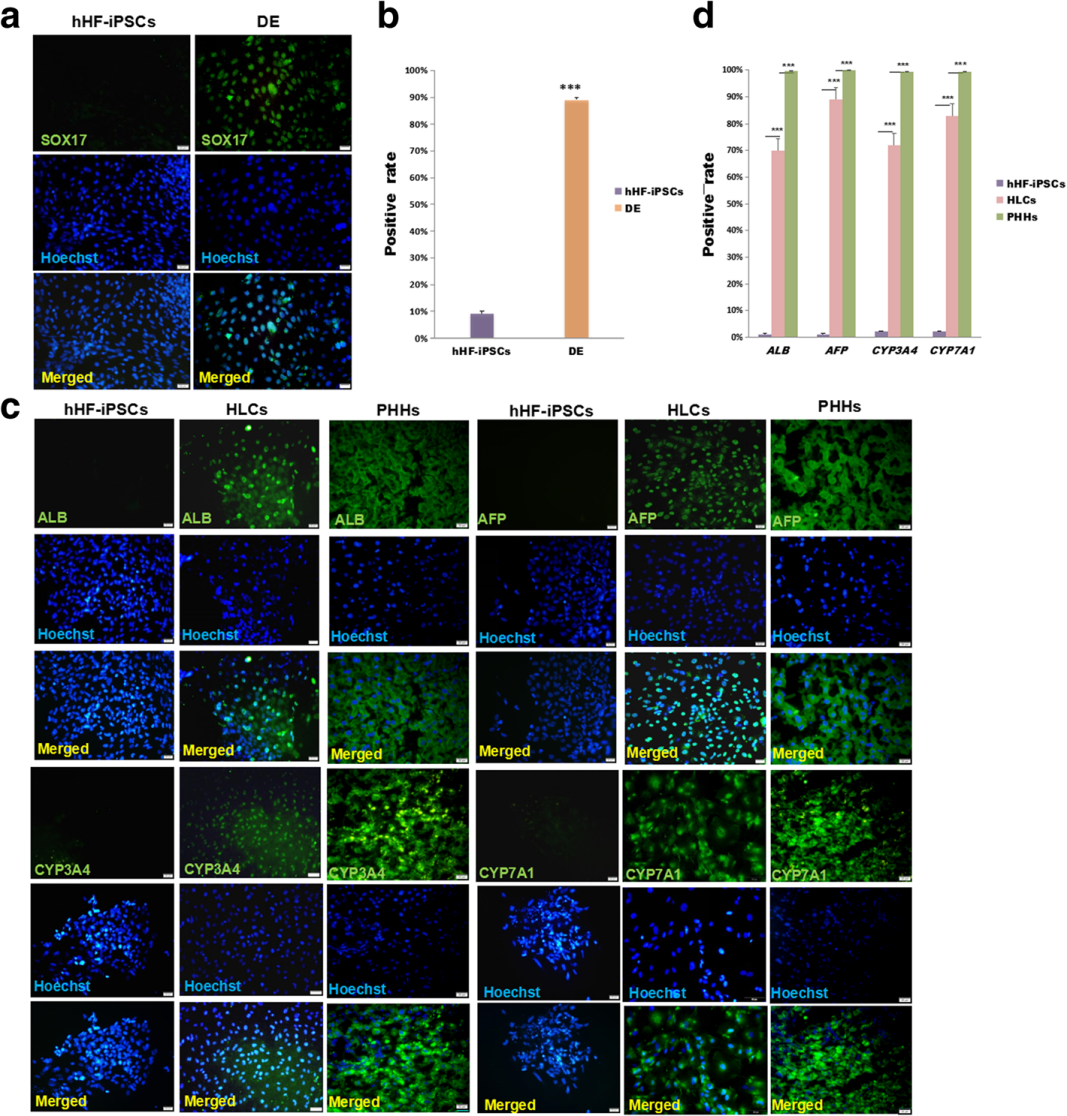
Protein levels in DE, HLCs, and PHHs. **a** Protein levels in DE. hHF-iPSCs and DE immunostained for SOX17 (green) and counterstained with DAPI (blue). **b** Quantification of SOX17 expression in hHF-iPSCs and DE (***P < 0.001; *n* ≥ 3). **c** Protein levels in HLCs. hHF-iPSCs, HLCs, and PHHs immunostained for ALB, AFP, CYP3A4, and CYP7A1 (green), and counterstained with DAPI (blue) **d** Quantification of ALB, AFP, CYP3A4, and CYP7A1 expression in hHF-iPSCs, HLCs, and PHHs (***P < 0.001; n ≥ 3). Scale bars = 20 μm. AFP alpha-fetoprotein, ALB albumin, CYP cytochrome P450, DE definitive endoderm, hHF-iPSC human hair follicle-derived induced pluripotent stem cell, HLC hepatocyte-like cell, PHH primary human hepatocyte

During days 1–4, hHF-iPSCs dispersed gradually (Fig. 1a). On day 5, the DE was formed [19] (Fig. 1b). To quantify the protein expression level of the DE, immunofluorescence staining was used to detect the expression of specific marker SOX17, as an early pan-endodermal transcription factor that is limited later in the DE during embryogenesis [20]. DE cells showed apparent specificity for expression of SOX17 protein (Fig. 2a). The statistical analysis showed that the positive rate of SOX17 was significantly increased to 89% from 9% (*P* < 0.001; *n* ≥ 3) (Fig. 2b). This showed that the method of DE induction was correct and feasible.

During days 5–19, the cell morphology changed significantly, and cells displayed a cobble-stone morphology with outgrowth and were stereo similar to the morphology of normal human hepatocytes [21] (Fig. 1c). On day 19, the cells were called HLCs [4]. Compared with the hHF-iPSCs, after induction for 19 days, hHF-iPSC-derived HLCs showed strong expression of ALB, AFP, CYP3A4, and CYP7A1; hHF-iPSCs did not express ALB and AFP, while HLCs expressed ALB and AFP strongly. hHF-iPSCs expressed CYP3A4 and CYP7A1 weakly, while HLCs expressed these markers at significantly higher levels. The expression of ALB, AFP, CYP3A4, and CYP7A1 in hepatic tissue was 100% (Fig. 2c). Statistical analysis confirmed that the difference in ALB, AFP, CYP3A4, and CYP7A1 expression between HLCs and hHF-iPSCs was significant, proving that we obtained the HLCs with high expression of ALB, AFP, CYP3A4 and CYP7A1, but the expression of ALB, AFP, CYP3A4, and CYP7A1 in HLCs still showed a significant difference compared with PHHs (*P* < 0.001; *n* ≥ 3) (Fig. 2d).

### Functional identification of HLCs

The liver is an important organ for detoxification of the human body. Mature hepatocytes have various functions, such as glycogen synthesis and storage, metabolism, and excretion. Therefore, in addition to morphology and protein expression, we also tested whether hHF-iPSC-derived HLCs had functional characteristics similar to mature hepatocytes.

**Fig. 3:**
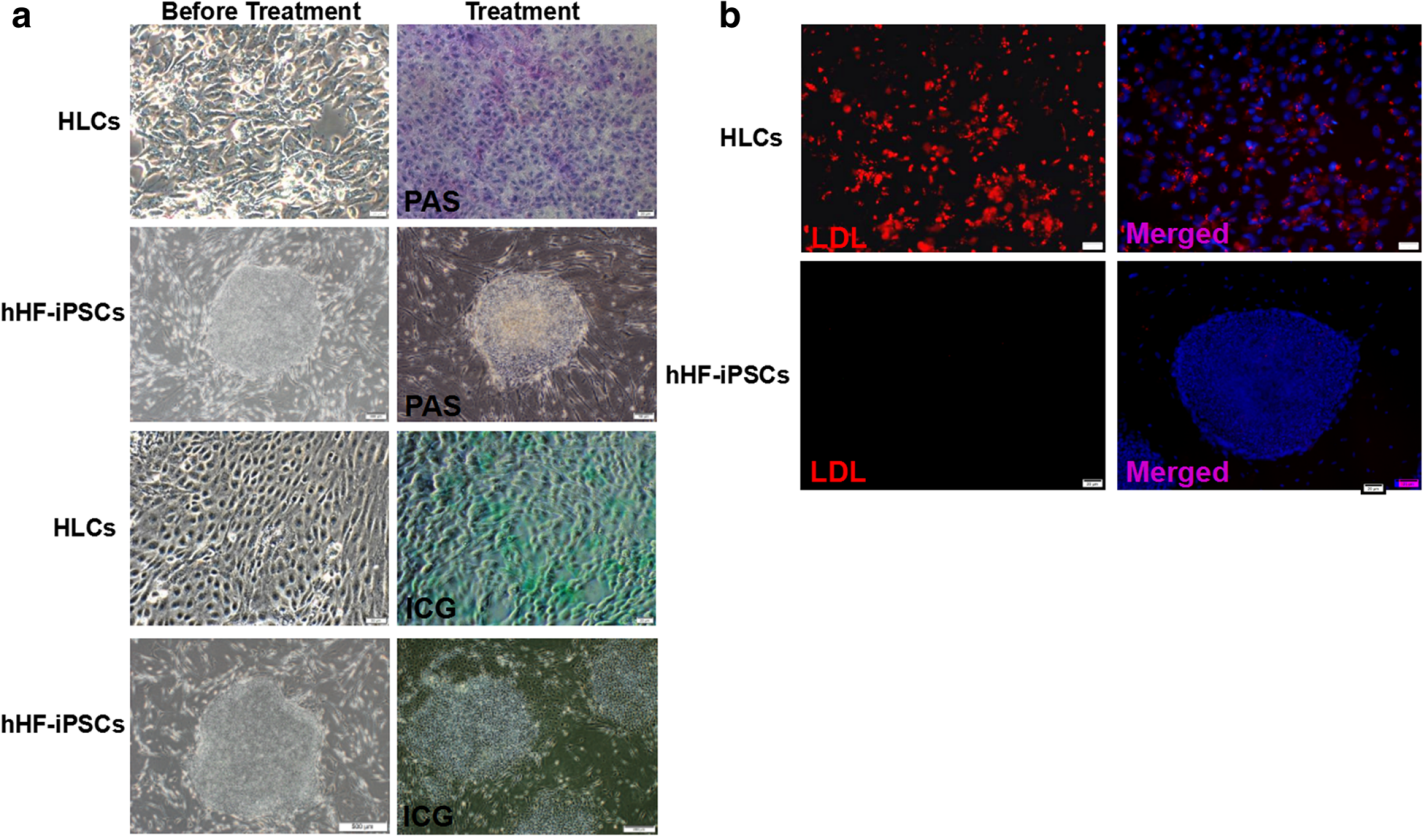
Functional identification of HLCs. **a** Glycogen storage function detected in hHF-iPSCs and HLCs by PAS (red). Transport and metabolic functions of hHF-iPSCs and HLCs evaluated by ICG (green) uptake and release. **b** LDL uptake detected in hHF-iPSCs and HLCs by staining with LDL-Dylight 550 (red) and counterstained with DAPI (blue). Scale bars = 20 μm. hHF-iPSC human hair follicle-derived induced pluripotent stem cell, HLC hepatocyte-like cell, ICG indocyanine green, LDL low-density lipoprotein, PAS Periodic acid–Schiff

The synthesis and storage of glycogen is an important function of hepatocytes. In this experiment, we analyzed the glycogen storage capability of HLCs by the Schiff method (PAS staining). Red granular material was visible (Fig. 3a), indicating positive PAS staining and proving that the HLCs were capable of performing glycogen synthesis and storage function. Additionally, we conducted ICG excretion and uptake experiments to detect the discharge capacity of HLCs. In the ICG excretion and uptake experiments, the presence of green material was observed, which proved that the HLCs possessed the ability for ICG uptake and excretion. In contrast, hHF-iPSCs were negative for PAS and did not harbor any green material, indicating that they did not possess the properties of glycogen storage and ICG excretion and uptake (Fig. 3a). Next, we explored the metabolic function of HLCs using the LDL uptake experiment. In the LDL uptake experiments, we found a strong red fluorescence signal specific for LDL in HLCs (Fig. 3b), suggesting that our induction conferred HLCs with LDL uptake ability, while LDL uptake was not observed in hHF-iPSCs.

The functional experiment proved our induction scheme could promote hHF-iPSCs to differentiate into HLCs and that these HLCs were capable of performing storage and metabolism functions.

### Identification of DEGs

**Fig. 4:**
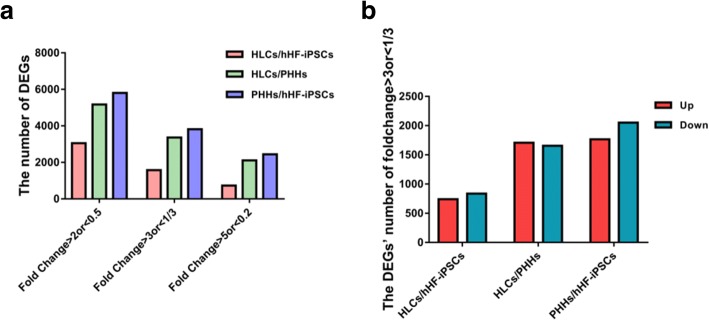
Comparison of number of DEGs. **a** Number of DEGs with fold change > 2 or < 0.5, > 3 or < 1/3, and > 5 or < 0.2 in HLCs/hHF-iPSCs, HLCs/PHHs, and PHHs/hHF-iPSCs. **b** DEGs with fold change > 3 or < 1/3 in HLCs/hHF-iPSCs, HLCs/PHHs, and PHHs/hHF-iPSCs. DEGs differentially expressed genes, hHF-iPSC human hair follicle-derived induced pluripotent stem cell, HLC hepatocyte-like cell, PHH primary human hepatocyte

The technical route of the data analysis series is shown in Fig. 1d. In order to further investigate the gene expression of hHF-iPSCs, HLCs, and PHHs, we applied the full transcriptional chip HTA2.0 from Affymetrix and generated the gene expression profile of hHF-iPSCs, induced HLCs, and PHHs. In this process, a total of 67,528 probes were used after removing duplicate probes and 26,730 genes were obtained. After induction for 20 days in vitro, a complete list of identified genes of HLCs obtained was available on HTA2.0. The numbers of DEGs for HLCs/hHF-iPSCs, HLCs/PHHs, and PHHs/hHF-iPSCs are presented in Table 1. Change in gene expression was measured in terms of fold change: fold change = 2^(former expression value – latter expression value)^. Regardless of the extent of the fold change, the number of DEGs was greatest in PHHs/hHF-iPSCs. However, hHF-iPSCs differentiated into functional hepatocytes despite there being a significantly minimal number of DEGs between hHF-iPSCs and the other two groups. For a more direct comparison, we generated a histogram of the total number of DEGs among the three groups (Fig. 4a). Considering the number of DEGs and the fold change, we chose to analyze the DEGs, both upregulated and downregulated, showing fold change > 3 or < 1/3 (Fig. 4b). Considering upregulated or downregulated DEGs, the number of DEGs in PHHs/hHF-iPSCs was greater than that in HLCs/hHF-iPSCs.

**Table 1:** Numbers of DEGs for the three cell groups

Group	Fold Change > 2	Fold change < 0.5	Fold change > 3	Fold change < 1/3	Fold change > 5	Fold change < 0.2
HLCs/hHF-iPSCs	1326	1726	741	837	399	332
HLCs/PHHs	2720	2451	1707	1656	993	1116
PHHs/hHF-iPSCs	2635	3170	1766	2052	1206	1234

*hHF-iPSC* human hair follicle-derived induced pluripotent stem cell, *HLC* hepatocyte-like cell, *PHH* primary human hepatocyte

In the HLCs/hHF-iPSCs group, the 10 downregulated DEGs were mainly associated with somatic stem cell population maintenance, such as *NANOG*, *PRDM14*, *SOX2*, and *TDGF1* (Additional file 1: Table S1). For the HLCs/PHHs group, most of the 10 downregulated DEGs were related to the acute-phase response, such as *CRP*, *APCS*, *HP*, and *ORM2* (Additional file 1: Table S2). For the PHHs/hHF-iPSCs group, the upregulated DEGs *ALB*, *APOH*, *FGA*, *FGG*, and *ORM2* were enriched in platelet degranulation, and *CRP*, *APCS*, *HP*, and *ORM2* were enriched in the acute-phase response. In contrast, the 10 downregulated DEGs such as *GJA1* and *VCAN* were enriched in osteoblast differentiation (Additional file 1: Table S3). These results revealed that the pluripotency of cells decreased during our induction process. However, the expression of DEGs related to the metabolic function in HLCs was lower than that in PHHs.

### GO term enrichment analysis

The GO analysis results between HLCs and hHF-iPSCs showed that the upregulated DEGs were significantly enriched in the BP, including development of cardiovascular, circulatory system, and cell migration (Additional file 1: Table S4); the downregulated DEGs were significantly enriched in the BP, including chromatin silencing in rDNA, chromosome organization, and negative regulation of DEG expression (Additional file 1: Table S4). The CC analysis also showed that the upregulated DEGs were significantly enriched in the extracellular matrix, proteinaceous extracellular matrix, and extracellular matrix component, whereas the downregulated DEGs were enriched in the chromosome and nuclear chromosome (Additional file 1: Table S4). In addition, in the MF analysis, the upregulated DEGs were enriched in growth factor binding and cell adhesion molecule binding, and the downregulated DEGs were enriched in heterocyclic compound binding, organic cyclic compound binding, and nucleic acid binding (Additional file 1: Table S4).

The GO analysis results between HLCs and PHHs showed that the upregulated DEGs were significantly enriched in the BP, including mitotic cell cycle, cell cycle processes, and chromosome organization (Additional file 1: Table S5); the downregulated DEGs were significantly enriched in biological processes, including organic, carboxylic, and monocarboxylic acid metabolic processes (Additional file 1: Table S5). Additionally, the CC analysis revealed that the upregulated DEGs were significantly enriched in the chromosomal region, anchoring junction, and extracellular matrix, and the downregulated DEGs were enriched in extracellular exosomes and vesicles (Additional file 1: Table S5). In addition, in the MF, the upregulated DEGs were enriched in macromolecular complex and cell adhesion molecule binding, and the downregulated DEGs were enriched in monooxygenase activity, cofactor binding, and oxidoreductase activity, acting on the CH–OH group of donors (Additional file 1: Table S5).

The GO analysis results between PHHs and hHF-iPSCs showed that upregulated DEGs were significantly enriched in the BP, including organic acid, oxoacid, and carboxylic acid metabolic process (Additional file 1: Table S6); the downregulated DEGs were significantly enriched in biological processes, including chromosome organization, mitotic cell cycle, and cell cycle processes (Additional file 1: Table S6). Additionally, the CC analysis revealed that the upregulated DEGs were significantly enriched in the extracellular exosome, vesicle, and organelle, whereas the downregulated DEGs were enriched in the nucleoplasm, chromosomal region, and nuclear chromosome (Additional file 1: Table S6). Additionally, in the MF, the upregulated DEGs were enriched in monooxygenase and oxidoreductase activity, acting on paired donors, with incorporation or reduction of molecular oxygen, and the downregulated DEGs were enriched in nucleic acid, heterocyclic compound, and organic cyclic compound binding (Additional file 1: Table S6).

**Fig. 5:**
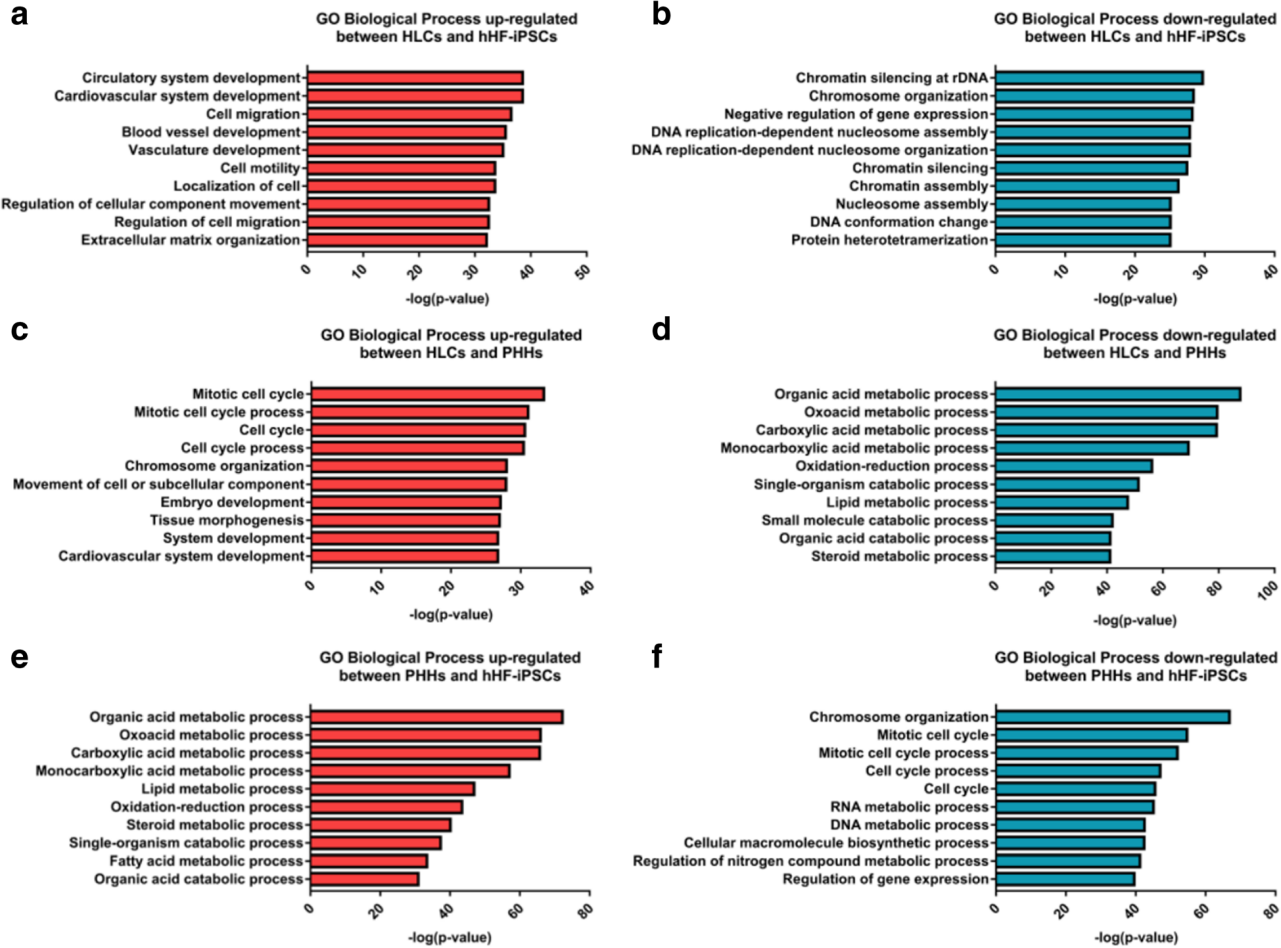
GO biological process for upregulated and downregulated DEGs of (**a**, **b**) HLCs/hHF-iPSCs, (**c**, **d**) HLCs/PHHs, and (**e**, **f**) PHHs/hHF-iPSCs, respectively. GO Gene Ontology, hHF-iPSC human hair follicle-derived induced pluripotent stem cell, HLC hepatocyte-like cell, PHH primary human hepatocyte

We generated a list of the DEGs with the top 10 *P* values according to the GOTERM_BP_FAT list for biological processes of general interest (in line with the Benjamini ranking). After comparing the three groups, it was found that the enrichment of DEGs related to cardiovascular system development in the HLCs/hHF-iPSCs and HLCs/PHHs groups was upregulated, indicating that the enrichment level of DEGs related to the cardiovascular system development in HLCs was too high. The enrichment of chromosome organization-related DEGs in HLCs/hHF-iPSCs as well as PHHs/hHF-iPSCs was downregulated, while it was upregulated in HLCs/PHHs; our induction process for this DEG enrichment was effective. Also, the enrichment of downregulated DEGs in the HLCs/PHHs group was almost the same as that of upregulated DEGs in the PHHs/hHF-iPSCs group; in addition, the upregulated DEG enrichment in HLCs/PHHs occurred in the mitotic cell cycle, mitotic cell cycle process, cell cycle, and cell cycle process, and chromosome organization was downregulated in the PHHs/hHF-iPSCs group (Fig. 5).

### KEGG pathway analysis

**Fig. 6:**
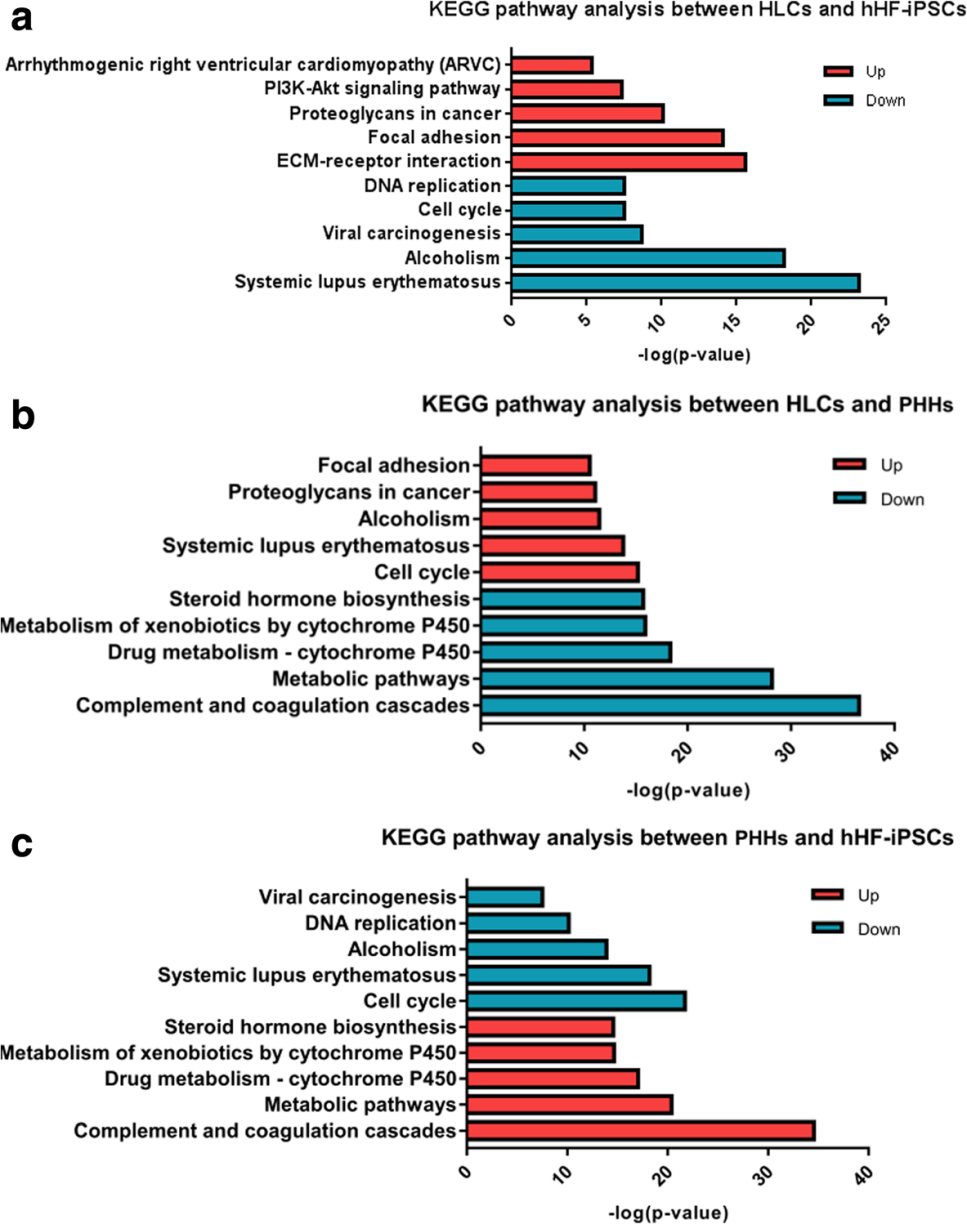
KEGG pathway analysis for (**a**) HLCs/hHF-iPSCs, (**b**) HLCs/PHHs, and (**c**) PHHs/hHF-iPSCs. KEGG pathways of upregulated DEGs (red) and downregulated DEGs (blue) analyzed. ECM extracellular matrix, hHF-iPSC human hair follicle-derived induced pluripotent stem cell, HLC hepatocyte-like cell, KEGG Kyoto Encyclopedia of Genes and Genomes, PHH primary human hepatocyte

The most significantly enriched pathways of the upregulated and downregulated DEGs in the KEGG analysis conducted on DAVID are shown in Fig. 6. Between HLCs and hHF-iPSCs, the upregulated DEGs were enriched in ECM-receptor interaction, focal adhesion, proteoglycans in cancer, PI3K–Akt signaling pathway, and arrhythmogenic right ventricular cardiomyopathy (ARVC) pathways, while the downregulated DEGs were enriched in systemic lupus erythematosus, alcoholism, viral carcinogenesis, cell cycle, and DNA replication. Between HLCs and PHHs, the upregulated DEGs were enriched in cell cycle, systemic lupus erythematosus, alcoholism, proteoglycans in cancer, and focal adhesion pathways, while the downregulated DEGs were enriched in complement and coagulation cascades, metabolic pathways, drug metabolism-cytochrome P450, metabolism of xenobiotics by cytochrome P450, and steroid hormone biosynthesis. Between PHHs and hHF-iPSCs, the upregulated DEGs were enriched in complement and coagulation cascades, metabolic pathways, drug metabolism-cytochrome P450, metabolism of xenobiotics by cytochrome P450, and steroid hormone biosynthesis pathways, while the downregulated DEGs were enriched in cell cycle, systemic lupus erythematosus, alcoholism, DNA replication, and viral carcinogenesis. Specific information on the top five pathways in the KEGG pathway analysis for each group of upregulated and downregulated DEGs is listed in Additional file 1: Tables S7–S9. For the top five enrichments, the downregulated pathways of the HLCs/hHF-iPSCs group were consistent with the downregulated pathways of PHHs/hHF-iPSCs; however, alcoholism, systemic lupus erythematosus, and cell cycle were upregulated in the HLCs/PHHs group, and the upregulated pathways between HLCs/hHF-iPSCs and PHHs/hHF-iPSCs were inconsistent, while the downregulated pathways of the HLCs/PHHs group were consistent with the upregulated pathways of the PHHs/hHF-iPSCs group. This result indicated that HLCs were consistent with PHHs in the top five downregulated pathways but needed to be further downregulated for alcoholism, systemic lupus erythematosus, and cell cycle, and the upregulated pathways were quite different. The complement and coagulation cascades, metabolic pathways, drug metabolism-cytochrome P450, metabolism of xenobiotics by cytochrome P450, and steroid hormone biosynthesis needed to be mainly increased (Fig. 6).

### FunRich site of expression analysis

**Fig. 7:**
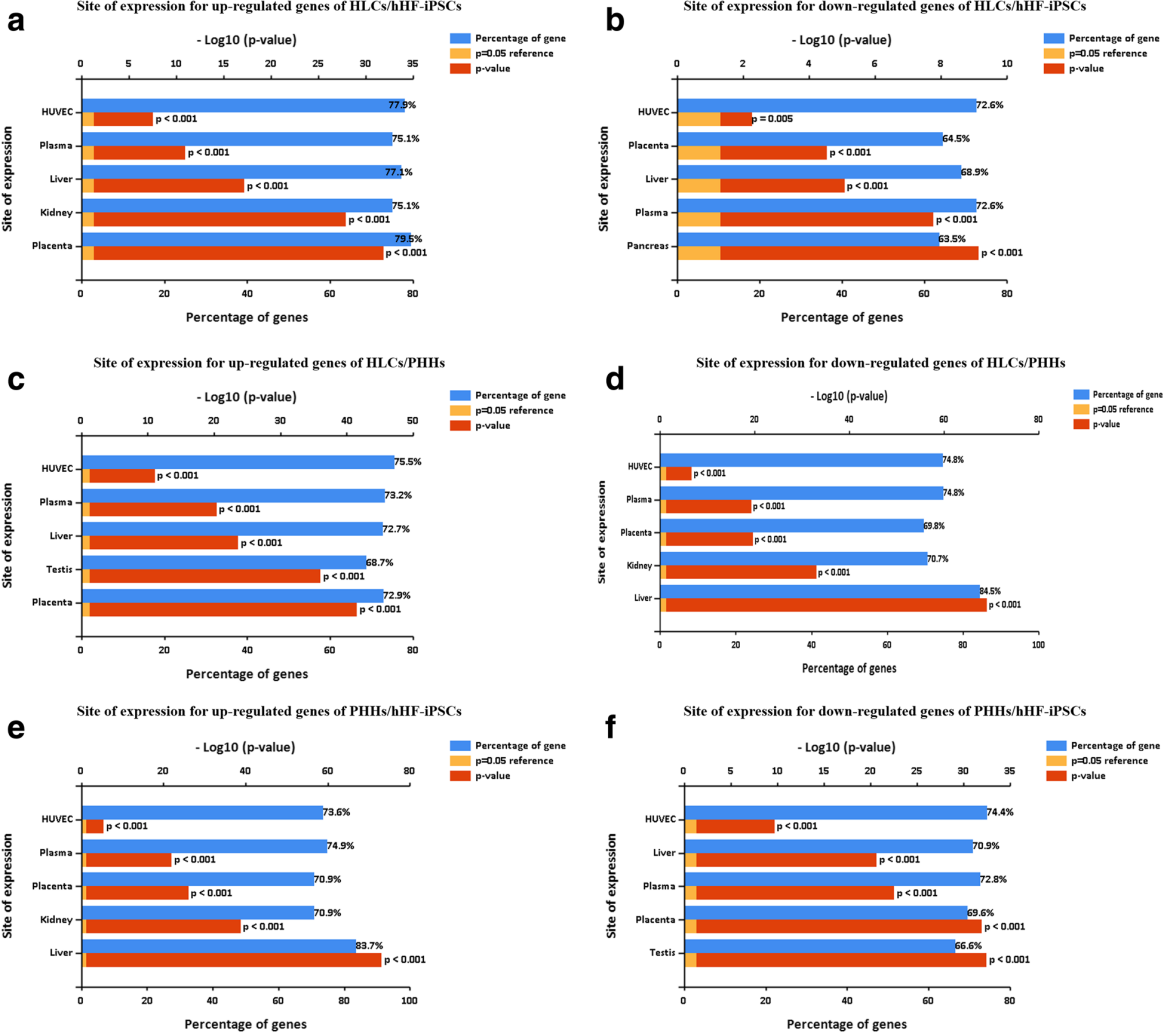
FunRich site of expression analysis for upregulated and downregulated DEGs of (**a**, **b**) HLCs/hHF-iPSCs, (**c**, **d**) HLCs/PHHs, and (**e**, **f**) PHHs/hHF-iPSCs, respectively. hHF-iPSC human hair follicle-derived induced pluripotent stem cell, HLC hepatocyte-like cell, PHH primary human hepatocyte

To further analyze the site of expression, we selected FunRich software, which is completely free and easy to use. The results of this analysis are shown in Fig. 7. This analysis included the various upregulated and downregulated DEGs of these three groups. We obtained the gene rankings based on the percentage of DEGs after FunRich software analysis and exported the top five DEGs. The output pictures were automatically ranked according to –log_10_(*P* value). The results showed HUVECs, plasma, liver, and placenta in all sites of expression, indicating that the human HLCs we obtained are not of a single type but the cell group was closer to that of an organ in the transitional state. Except for those four kinds of the same sites of expression, the downregulated site of expression was the pancreas in HLCs/hHF-iPSCs, while the downregulated site of expression was the testis in PHHs/hHF-iPSCs, which was the only different place (Fig. 7).

### Integration of PPI network and module analysis

**Fig. 8:**
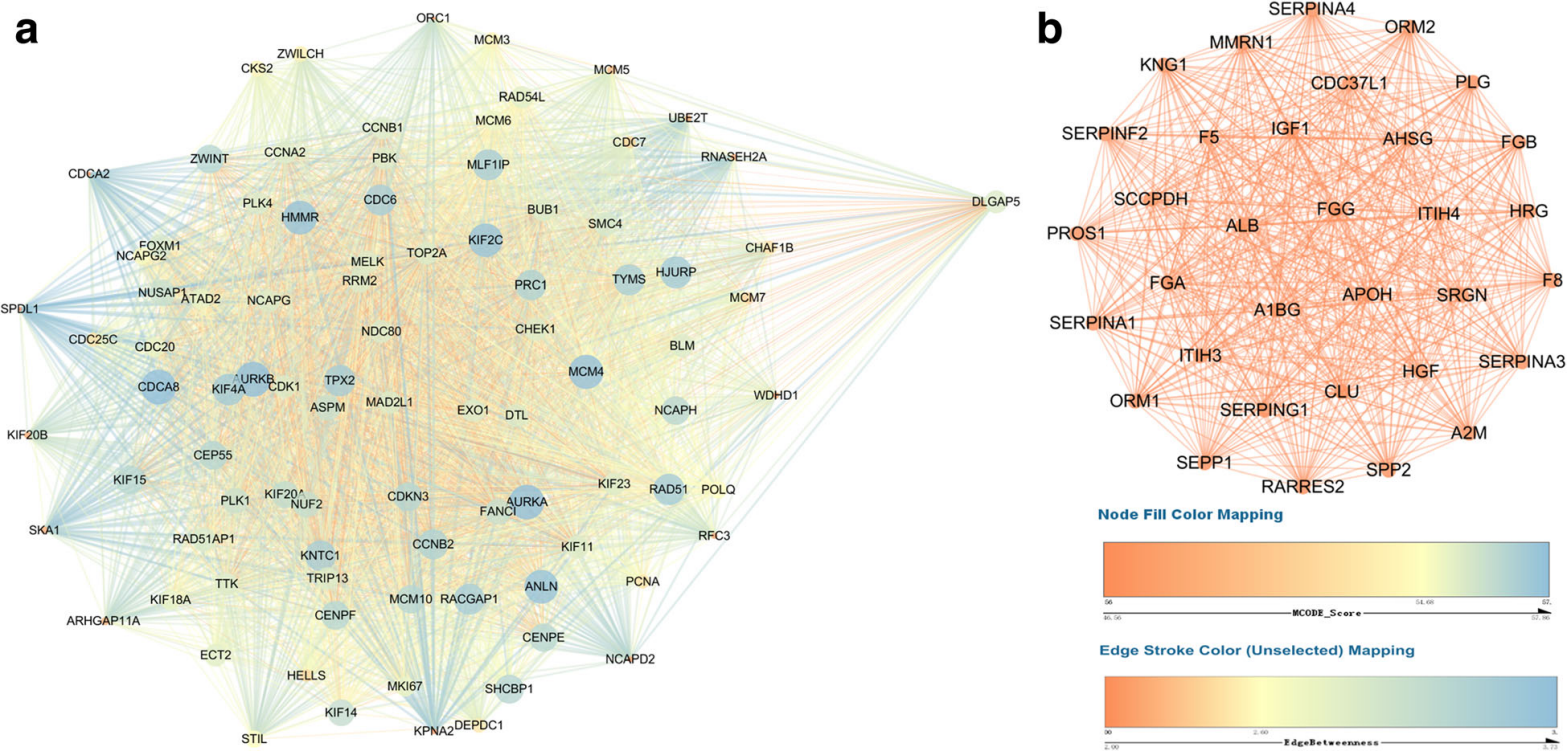
Hub cluster of DEG PPI network and module analysis in HLCs/PHHs. **a** MCODE score of upregulated cluster was 79.178. **b** MCODE score of downregulated cluster was 33. Different colors, node size, and edge width show the node’s MCODE score and the edge’s edge-betweenness

In order to compare the differences between induced HLCs and PHHs, we conducted PPI network and module analysis for shuffling and found new hub genes to provide new ideas for further improvement of the induction scheme. Based on the information in the STRING database, cytoscape software was used to generate a PPI network for the upregulated and downregulated DEGs and was analyzed according to the degree and MCODE score (Additional file 2). The top five upregulated DEGs were *TOP2A*, *CDK1*, *CCNB1*, *MYC*, and *PCNA*, while the top five DEGs with the highest degree of downregulation were *ALB*, *ACACB*, *DECR1*, *DPYD*, and *EHHADH* (Table 2). Compared with those in PHHs, in the HLCs the major upregulated DEGs were related to the cell cycle and the downregulated DEGs were related to metabolic processes. Then, we used MCODE to filter the subnetwork with the highest enrichment score and obtained an upregulated cluster of MCODE score 79.178 (Fig. 8a) and a downregulated cluster of MCODE score 33 (Fig. 8b); different colors, node size, and edge width showed the node’s MCODE score and edge-betweenness. It was found that the ranking of MCODE score genes in the cluster was consistent with the ranking of MCODE score genes in all DEG networks. The upregulated cluster contained 91 genes and the top 10 MCODE score genes were *AURKA*, *AURKB*, *CDCA8*, *MCM4*, *ANLN*, *HMMR*, *KIF2C*, *HJURP*, *KIF4A*, and *RAD51* (Table 3). Compared with those in PHHs, in the HLCs the major upregulated DEGs were related to oocyte meiosis, cell cycle, ECM-receptor interaction, and pathways in cancer. The downregulated cluster comprised 33 genes and they had the same degree and MCODE score of 33; therefore, we focused on the top 10 upregulated MCODE score genes.

**Table 2:** Top 10 genes for degree in PPI network for DEGs between HLCs and PHHs

Gene	Degree	Top *P*-value KEGG pathway	Top *P*-value GOTERM_BP_FAT	Fold change
Upregulated DEGs				
*TOP2A*	260	None	GO:0000278: mitotic cell cycle	218.0750636
*CDK1*	235	hsa04110: cell cycle	GO:0000278: mitotic cell cycle	11.94033162
*CCNB1*	198	hsa04110: cell cycle	GO:0000278: mitotic cell cycle	30.20813056
*MYC*	196	hsa04110: cell cycle	GO:0007049: cell cycle	6.04915656
*PCNA*	193	hsa04110: cell cycle	GO:0000278: mitotic cell cycle	5.543822244
Downregulated DEGs				
*ALB*	250	None	GO:0010876: lipid localization	9.20E-05
*ACACB*	151	hsa01100: metabolic pathways	GO:0006082: organic acid metabolic process	0.009806799
*DECR1*	138	None	GO:0006082: organic acid metabolic process	0.159393422
*DPYD*	128	hsa01100: metabolic pathways	GO:0006082: organic acid metabolic process	0.015090098
*EHHADH*	115	hsa01100: metabolic pathways	GO:0006082: organic acid metabolic process	0.020681003

*DEG* differentially expressed gene, *GO* Gene Ontology, *HLC* hepatocyte-like cell, *PHH* primary human hepatocyte, *KEGG* Kyoto Encyclopedia of Genes and Genomes, *PPI* protein–protein interaction

**Table 3:** Top 10 genes for MCODE score in PPI network for upregulated DEGs between HLCs and PHHs

Gene	MCODE score	Top *P*-value KEGG pathway	Top *P*-value GOTERM_BP_FAT	Fold change
*AURKA*	57.86384977	hsa04114: oocyte meiosis	GO:0000278: mitotic cell cycle	3.351277145
*AURKB*	57.86384977	None	GO:0000278: mitotic cell cycle	3.114371397
*CDCA8*	57.86384977	None	GO:0000278: mitotic cell cycle	4.028863704
*MCM4*	57.74647887	hsa04110: cell cycle	GO:0000278: mitotic cell cycle	10.1500716
*ANLN*	57.67123288	None	GO:0000278: mitotic cell cycle	201.1978391
*HMMR*	57.67123288	hsa04512: ECM-receptor interaction	GO:0000278: mitotic cell cycle	9.742803711
*KIF2C*	57.64840183	None	GO:0000278: mitotic cell cycle	7.390090337
*HJURP*	57.28985507	None	GO:0007049: cell cycle	3.334170774
*KIF4A*	57.28985507	None	GO:0000278: mitotic cell cycle	8.522401381
*RAD51*	57.24546464	hsa05200: pathways in cancer	GO:0007049: cell cycle	3.486689757

*DEG* differentially expressed gene, *ECM* extracellular matrix, *GO* Gene Ontology, *HLC* hepatocyte-like cell, *PHH* primary human hepatocyte, *KEGG* Kyoto Encyclopedia of Genes and Genomes, *PPI* protein–protein interaction

## Discussion

Shi et al. [5] found that reprogrammed hHF-iPSCs showed the potential to directly differentiate into functional hepatocytes. We induced hHF-iPSCs into HLCs via four stages (Fig. 1). Quantitative analysis indicated that using B27 could effectively induce the differentiation of hiPSCs into endoderm cells [22]. A few studies suggested that activin A could be used in the process of forming endoderm rows [23, 24]. Hay et al. [25] used activin A combined with Wnt3a to induce liver endoderm cells of relatively consistent form and could play the function of hepatocytes more efficiently than other cytokine combinations. *SOX17* is very important in embryonic development and is an endoderm marker; therefore, *SOX17* was also expressed at low levels during development, and would increase significantly in the endoderm [20]. FGFs derived from cardiac mesoderm and BMPs derived from mesenchymal cells in the lateral compartment have been shown to be essential for the induction of endoderm cells into hepatocytes [26, 27]. Cai et al. [28] found that FGF-4 alone and BMP-2 alone had little effect on the culture of endoderm cells; however, both of these lead to a significant increase in the number of cells expressing *ALB* in vitro, and they also demonstrated that the induction of FGF-4 and BMP-2 was the key to efficient induction of hepatocytes by the endoderm. OSM had been reported to promote differentiation of progenitor cells into hepatocytes [29]. HGF enhances the maturation and proliferation of hepatocytes [30], and DEX is a synthetic glucocorticoid that is involved in gluconeogenesis [31]. Through these factors in the four stages, the hHF-iPSCs could differentiate into functional HLCs. *AFP* is an early hepatic marker, while ALB is the most abundant protein synthesized by hepatocytes [32]; additionally, most *CYPs* (including *CYP3A4*, *CYP7A1*, and *CYP2D6*) are only slightly expressed or not detected in the fetal liver tissue [33], and we thus chose *ALB*, *AFP*, *CYP3A4*, and *CYP7A1* as hepatocyte markers.

How the function of HLCs can be improved remains to be further studied. Based on our data, the hub of PPI network for upregulated DEGs showed that there was a potential cancer risk in the process of differentiation of hHF-iPSCs into HLCs; how to reduce the risk of cancer and make HLC function more consistent with that of PHHs is the next experimental target. In order to compare the gaps between HLCs and PHHs, and to provide a theoretical basis for solving the problems in the induction process for future research, we performed a genome-wide transcriptome analysis of hHF-iPSCs, HLCs, and PHHs, and obtained DEGs among the three groups.

In order to further judge the differences and optimize the solution, we conducted the following analysis. Through analysis of the top 10 upregulated and downregulated DEGs of the three groups and using DAVID to view the enrichments of these DEGs, the genes of stem cell pluripotency in HLCs/hHF-iPSCs were downregulated, such as *TDGF1*, *PRDM14* [34], *SOX2*, and *NANOG* [35]. This showed that we induced differentiation of hHF-iPSCs; however, compared with PHHs, HLCs were found to upregulate DEGs associated with acute phase reactions such as *APCS*, *ORM2* [36], *HP*, and *CRP*.

To better understand the function of DEGs, we analyzed the upregulated and downregulated DEGs among hHF-iPSCs, HLCs, and PHHs by GO and KEGG pathway enrichment analysis. GO analysis showed that compared to hHF-iPSCs, upregulated DEGs of PHHs majorly involved the metabolic process of organic acid, oxoacid, carboxylic, etc., and the downregulated DEGs majorly involved in the chromosome organization, mitosis, and cell cycle processes. This is consistent with the main factors and progression of the stem cells differentiating to hepatocytes. HLCs/hHF-iPSCs and HLCs/PHHs indicated that the downregulation of DEGs regulating chromosome organization was appropriate for induction of hHF-iPSCs to form HLCs; however, the cardiovascular system-related genes in HLCs were quite highly expressed. In addition, the mitotic cell cycle and cell cycle genes were expressed at high levels; however, the expression of genes involved in the metabolism of organic acid, oxoacid, carboxylic, etc. was low, indicating that the HLCs were insufficient in these aspects and the induction program needed to be adjusted. In addition, for the KEGG pathway enrichment analysis, the enriched KEGG pathways of upregulated DEGs in PHHs/hHF-iPSCs included complement and coagulation cascades, metabolic pathways, drug metabolism-cytochrome P450, and other biosynthesis as well as metabolic pathways. Downregulated DEGs were associated with cell cycle, systemic lupus erythematosus, alcoholism, DNA replication, and viral carcinogenesis pathways, indicating that differentiation reduced the cell proliferation and tumorigenicity risk. Additionally, from the enriched KEGG pathways of upregulated DEGs in HLCs/hHF-iPSCs and HLCs/PHHs groups, we found that the induced HLCs further repressed the downregulated pathways, consistent with the PHHs/hHF-iPSCs group; however, the pathways of alcoholism, cell cycle, and systemic lupus erythematosus continued to be downregulated, and the complement and coagulation cascades, metabolic pathways, drug metabolism-cytochrome P450, and steroid hormone biosynthetic pathway-associated DEGs needed to be increased [8]. Although our experiments confirmed that the HLCs we obtained mimicked the synthesis and metabolism functions of the liver, our data analysis supported that they needed to be further improved.

FunRich is a novel community-driven approach to build a comprehensive functional enrichment analysis tool [15]. Using the FunRich site of expression analysis, we speculated that the HLCs were not pure cells; but various components such as HUVECs, plasma, liver, and placenta which were similar to those in liver organoids. Although organoids are not human organs in the true sense, they can mimic the structure and function of true organs, and can be rapidly constructed. Organoids which play an important role in the research on human organ development, disease mechanisms, drug screening, and organ repair materials are likely to provide valuable tools for revealing disease mechanisms, designing novel and personalized treatment strategies, and generating autologous stem cells for gene editing and transplantation purposes [37]. There have been studies using liver-like organs to construct disease models, such as fatty liver [38]. Additionally, HLCs have been applied to study HCV infection [39], and show promise for use as liver organs for further research.

PPI network and module analysis was constructed for the HLC/PHHs group, and we found that the ranking of MCODE score genes in the cluster was consistent with the ranking of MCODE score genes in all DEG networks; however, the degree of ranking was different between the cluster and the network, so we analyzed the top five upregulated and downregulated DEGs. The hub cluster of downregulated DEGs had 33 genes of the same degree and MCODE score, but the upregulated hub cluster had genes with a different MCODE score. The top five upregulated DEGs, including *CDK1*, *CCNB1* [40], *TOP2A* [41], *MYC* [42], and *PCNA* [43], were associated with cell mitosis and involved in the development of cancer. Additionally, *CDK1* was analyzed as the gene of the highest degree in the DEG PPI network of iPSCs and hepatocytes [44]. Furthermore, it was reported that *CDK1* could regulate the self-renewal and differentiation of hESCs [45]. Most of the top five downregulated DEGs (*ALB*, *ACACB*, *DECR1*, and *EHHADH*) were associated with fatty acid biosynthesis [46]; however, *DPYD* encodes a gene for two-hydrogen pyrimidine dehydrogenase (DPD). DPD was one of the metabolic anticancer drugs, fluorouracil (5-Fu), in the liver [47]. The top 10 genes of the MCODE score included *AURKA*, *AURKB*, *CDCA8*, and *MCM4*. Among these, *CDCA8* (cell division cycle associated 8) activated *AURKB* (Aurora B kinase) and played an important role in liver regeneration and development of tumor, and might be a potential target for related liver diseases [48]. In addition, the expression of *AURKA* was positively correlated to TP53 mutation in human liver cancer [49]. The gene expression of *MCM4* and *MCM6* was reduced in aging HSCs, and these are the two components which correctly replicate the necessary MCM protein complexes [50]. These could be the main gene targets for our optimized induction protocol, but we still need to experimentally verify whether these genes could acquire mature hepatocytes in our induction programs, and it was possible to acquire mature hepatocytes by upregulation of metabolism-related gene *ALB*, or downregulation of *CDK1*, *MCM4*, etc.

HLCs have been proved useful for disease modeling and cell therapy, but there are obvious differences between HLCs and PHHs [2, 51]. Our induction program can be used as a reference for liver organoids. We have conducted a comprehensive DEG bioinformatics analysis which provided many novel biomarkers, and targets for improving induction protocols and exploring molecular mechanisms for hepatic differentiation and liver organoid induction in the future. This study has clinical significance for drug screening and stem cell-based therapy for hepatic injuries; however, further molecular biological experiments are needed to confirm these findings.

## Conclusions

hHF-iPSCs could be successfully induced through the DE by addition of various stimulating factors at different stages of liver differentiation in vitro, and HLCs exhibited hepatocyte functions such as glycogen and low-density lipoprotein uptake. We found that the process of metabolism and synthesis in the HLC population should be strengthened. The expression of *TOP2A*, *CDK1*, etc. can be reduced or the expression of *ALB*, etc. can be increased to improve induction, and the roles of PI3K–Akt and metabolic pathways in the induction process need further exploration.

## Additional files

Additional file 1: **Table S1.**Top gene expression differences between HLCs and hHF-iPSCs. **Table S2.** Top gene expression differences between HLCs and PHHs. **Table S3.** Top gene expression differences between PHHs and hHF-iPSCs. **Table S4.** GO analysis of DEGs between HLCs and hHF-iPSCs. **Table S5.** GO analysis of DEGs between HLCs and PHHs. **Table S6.** GO analysis of DEGs between PHHs and hHF-iPSCs. **Table S7.** KEGG pathway analysis of DEGs between HLCs and hHF-iPSCs. **Table S8.** KEGG pathway analysis of DEGs between HLCs and PHHs. **Table S9.** KEGG pathway analysis of DEGs between PHHs and hHF-iPSCs. (DOCX 42 kb)

Additional file 2: PPI network and module analysis in HLCs/PHHs. (**A**) Network of all upregulated DEGs. (**B**) Network of all downregulated DEGs. Different colors, node size, and edge width show the node’s degree and the edge’s edge-betweenness. (JPG 64 kb)

## Abbreviations

ACT A: Activin A
AFP: Alpha-fetoprotein
ALB: Albumin
bFGF: Basic fibroblast growth factor
BMP: Bone morphogenetic protein
BP: Biological process
CC: Cell component
CYP: Cytochrome P450
DE: Definitive endoderm
DEG: Differentially expressed gene
DEX: Dexamethasone
DMSO: Dimethylsulfoxide
DPD: Two-hydrogen pyrimidine dehydrogenase
FBS: Fetal bovine serum
FGF: Fibroblast growth factor
GO: Gene Ontology
HGF: Hepatocyte growth factor
hHF-iPSC: Human hair follicle–derived iPSC
hiPSC: Human induced pluripotent stem cell
HLCs: Hepatocyte-like cells
HTA2.0: Human Transcriptome Array 2.0
ICG: Indocyanine green
KEGG: Kyoto Encyclopedia of Genes and Genomes
LDL: Low-density lipoprotein
MCODE: Molecular Complex Detection
MF: Molecular function
OSM: Oncostatin-M
PAS: Periodic acid–Schiff
PHH: Primary human hepatocyte
PPI: Protein–protein interaction
STRING: Search Tool for the Retrieval of Interacting Genes/Proteins
