# Supplementary material for: Analysis of differentially expressed genes among human hair follicle–derived iPSCs, induced hepatocyte-like cells, and primary hepatocytes
Source: Stem Cell Res Ther. 2018 Aug 9;9:211. doi: 10.1186/s13287-018-0940-z (PMC6085644; doi:10.1186/s13287-018-0940-z)
Supplement: Supplementary file 1 — Table S1.Top gene expression differences between HLCs and hHF-iPSCs. Table S2. Top gene expression differences between HLCs and PHHs. Table S3. Top gene expression differences between PHHs and hHF-iPSCs. Table S4. GO analysis of DEGs between HLCs and hHF-iPSCs. Table S5. GO analysis of DEGs between HLCs and PHHs. Table S6. GO analysis of DEGs between PHHs and hHF-iPSCs. Table S7. KEGG pathway analysis of DEGs between HLCs and hHF-iPSCs. Table S8. KEGG pathway analysis of DEGs between HLCs and PHHs. Table S9. KEGG pathway analysis of DEGs between PHHs and hHF-iPSCs. (DOCX 42 kb) [file 13287_2018_940_MOESM1_ESM.docx]

Table S1. Top Gene Expression Differences between HLCs and hHF-iPSCs

| **Gene** | **Expression in HLCs** | **Expression in hHF-iPSCs** | **Fold change** |
| --- | --- | --- | --- |
| **Top 10 upregulated DEGs** | | | |
| GABRP | 15.10665 | 5.261587 | 919.73 |
| ANKRD1 | 16.234 | 6.912363 | 639.87 |
| PRTG | 15.49364 | 6.532308 | 498.46 |
| HAND1 | 12.54706 | 3.67535 | 468.44 |
| EPAS1 | 13.60586 | 5.144152 | 352.56 |
| TMEM88 | 13.35496 | 5.875195 | 178.50 |
| COL3A1 | 13.06276 | 5.624252 | 173.47 |
| SERPINE1 | 15.35344 | 7.978249 | 166.02 |
| VTCN1 | 11.94124 | 4.603614 | 161.75 |
| TGFBI | 13.40283 | 6.116465 | 156.10 |
| **Top 10 downregulated DEGs** | | | |
| TDGF1 | 8.720168 | 15.18577 | 0.0113 |
| PRDM14 | 6.079042 | 12.63246 | 0.0106 |
| LOC100506507 | 5.517387 | 12.18515 | 0.0098 |
| SOX2 | 7.661105 | 14.37619 | 0.0095 |
| NANOG | 7.933583 | 14.65399 | 0.0095 |
| MIR1973 | 5.282052 | 12.06752 | 0.0091 |
| MT1G | 8.340573 | 15.17339 | 0.0088 |
| PTPRZ1 | 6.492453 | 13.38308 | 0.0084 |
| MT1H | 7.885231 | 14.87109 | 0.0079 |
| USP44 | 6.092518 | 13.34988 | 0.0065 |

Table S2. Top Gene Expression Differences between HLCs and PHHs

| **Gene** | **Expression in HLCs** | **Expression in PHHs** | **Fold change** |
| --- | --- | --- | --- |
| **Top 10 upregulated DEGs** | | | |
| ANKRD1 | 16.234 | 4.47447 | 3467.14 |
| PRTG | 15.49364 | 4.479966 | 2067.50 |
| SLC2A3 | 17.20789 | 7.204849 | 1026.16 |
| GABRP | 15.10665 | 5.261587 | 919.73 |
| MMP2 | 15.80234 | 6.551682 | 609.15 |
| CTGF | 15.35913 | 6.290479 | 536.95 |
| HAND1 | 12.54706 | 3.594357 | 495.49 |
| SLC7A5 | 14.75375 | 6.335784 | 342.03 |
| TMEM88 | 13.35496 | 5.157854 | 293.48 |
| CAPN6 | 12.86021 | 4.667317 | 292.62 |
| **Top 10 downregulated DEGs** | | | |
| F9 | 3.822846 | 16.75879 | 0.00013 |
| ALDOB | 4.705141 | 17.65286 | 0.00013 |
| APCS | 4.200751 | 17.3268 | 0.00011 |
| GC | 4.265417 | 17.57496 | 9.85E-05 |
| ORM2 | 4.596838 | 17.93857 | 9.63E-05 |
| ALB | 7.547701 | 17.95555 | 9.20E-05 |
| ADH1B | 4.287086 | 17.79508 | 8.58E-05 |
| HP | 4.334756 | 18.48976 | 5.48E-05 |
| CRP | 4.19728 | 18.45704 | 5.10E-05 |
| APOH | 3.978582 | 18.9362 | 3.14E-05 |

Table S3. Top Gene Expression Differences between PHHs and hHF-iPSCs

| **Gene** | **Expression in PHHs** | **Expression in hHF-iPSCs** | **Fold change** |
| --- | --- | --- | --- |
| **Top 10 upregulated DEGs** | | | |
| APOH | 18.9362 | 3.975587 | 31885.50 |
| CRP | 18.45704 | 4.212262 | 19413.57 |
| HP | 18.48976 | 4.677668 | 14383.15 |
| FGA | 18.61961 | 5.101427 | 11732.18 |
| ADH1B | 17.79508 | 4.289503 | 11630.11 |
| ALB | 17.95555 | 4.580639 | 10623.05 |
| GC | 17.57496 | 4.284745 | 10017.36 |
| APCS | 17.3268 | 4.227126 | 8777.98 |
| ORM2 | 17.93857 | 4.89642 | 8434.87 |
| FGG | 17.62706 | 4.611748 | 8279.41 |
| **Top 10 downregulated DEGs** | | | |
| GPC4 | 5.077828 | 14.09957 | 0.0019 |
| SLC7A3 | 5.10817 | 14.22265 | 0.0018 |
| SLC2A3 | 7.204849 | 16.41635 | 0.0017 |
| ZFP42 | 4.917987 | 14.31698 | 0.0015 |
| VCAN | 6.246413 | 15.67966 | 0.0014 |
| SLC7A11 | 4.21953 | 13.80079 | 0.0013 |
| DPPA4 | 5.378859 | 15.0652 | 0.0012 |
| DNMT3B | 6.151417 | 15.89136 | 0.0012 |
| ESRG | 6.8672 | 18.01052 | 0.0004 |
| GJA1 | 5.583993 | 16.87127 | 0.0004 |

Table S4. Gene ontology analysis of DEGs between HLCs and hHF-iPSCs

| **Expression** | **Category** | **Term** | **Gene count** | **％** | **P value** |
| --- | --- | --- | --- | --- | --- |
| Up-regulated | GOTERM_BP_FAT | GO:0072358~cardiovascular system development | 121 | 17.79 | 2.95E-39 |
|  | GOTERM_BP_FAT | GO:0072359~circulatory system development | 121 | 17.79 | 2.95E-39 |
|  | GOTERM_BP_FAT | GO:0016477~cell migration | 133 | 19.56 | 3.26E-37 |
|  | GOTERM_BP_FAT | GO:0001568~blood vessel development | 91 | 13.38 | 3.17E-36 |
|  | GOTERM_BP_FAT | GO:0001944~vasculature development | 93 | 13.68 | 8.89E-36 |
|  | GOTERM_CC_FAT | GO:0031012~extracellular matrix | 81 | 11.91 | 1.32E-29 |
|  | GOTERM_CC_FAT | GO:0005578~proteinaceous extracellular matrix | 65 | 9.56 | 9.72E-28 |
|  | GOTERM_CC_FAT | GO:0044420~extracellular matrix component | 38 | 5.59 | 3.62E-24 |
|  | GOTERM_CC_FAT | GO:0070161~anchoring junction | 79 | 11.62 | 6.03E-20 |
|  | GOTERM_CC_FAT | GO:0005925~focal adhesion | 57 | 8.38 | 9.24E-20 |
|  | GOTERM_MF_FAT | GO:0019838~growth factor binding | 30 | 4.41 | 1.96E-16 |
|  | GOTERM_MF_FAT | GO:0050839~cell adhesion molecule binding | 56 | 8.24 | 3.19E-16 |
|  | GOTERM_MF_FAT | GO:0005201~extracellular matrix structural constituent | 20 | 2.94 | 2.27E-11 |
|  | GOTERM_MF_FAT | GO:0005178~integrin binding | 22 | 3.24 | 4.88E-11 |
|  | GOTERM_MF_FAT | GO:0005539~glycosaminoglycan binding | 30 | 4.41 | 9.25E-11 |
| Down-regulated | GOTERM_BP_FAT | GO:0000183~chromatin silencing at rDNA | 28 | 3.74 | 1.95E-30 |
|  | GOTERM_BP_FAT | GO:0051276~chromosome organization | 127 | 16.96 | 4.11E-29 |
|  | GOTERM_BP_FAT | GO:0045814~negative regulation of gene expression, epigenetic | 44 | 5.87 | 6.01E-29 |
|  | GOTERM_BP_FAT | GO:0034723~DNA replication-dependent nucleosome organization | 25 | 3.34 | 1.42E-28 |
|  | GOTERM_BP_FAT | GO:0006335~DNA replication-dependent nucleosome assembly | 25 | 3.34 | 1.42E-28 |
|  | GOTERM_CC_FAT | GO:0005694~chromosome | 127 | 16.96 | 7.14E-39 |
|  | GOTERM_CC_FAT | GO:0044427~chromosomal part | 116 | 15.49 | 1.05E-36 |
|  | GOTERM_CC_FAT | GO:0000228~nuclear chromosome | 88 | 11.75 | 1.94E-31 |
|  | GOTERM_CC_FAT | GO:0032993~protein-DNA complex | 52 | 6.94 | 2.58E-31 |
|  | GOTERM_CC_FAT | GO:0044454~nuclear chromosome part | 84 | 11.21 | 1.29E-30 |
|  | GOTERM_MF_FAT | GO:1901363~heterocyclic compound binding | 331 | 44.19 | 1.37E-21 |
|  | GOTERM_MF_FAT | GO:0097159~organic cyclic compound binding | 333 | 44.46 | 3.36E-21 |
|  | GOTERM_MF_FAT | GO:0003676~nucleic acid binding | 250 | 33.38 | 8.73E-20 |
|  | GOTERM_MF_FAT | GO:0003677~DNA binding | 179 | 23.90 | 5.89E-19 |
|  | GOTERM_MF_FAT | GO:0046982~protein heterodimerization activity | 57 | 7.61 | 4.20E-15 |

Table S5. Gene ontology analysis of DEGs between HLCs and PHHs

| **Expression** | **Category** | **Term** | **Gene count** | **％** | **P value** |
| --- | --- | --- | --- | --- | --- |
| Up-regulated | GOTERM_BP_FAT | GO:0000278~mitotic cell cycle | 201 | 12.54 | 5.11E-34 |
|  | GOTERM_BP_FAT | GO:1903047~mitotic cell cycle process | 186 | 11.60 | 8.82E-32 |
|  | GOTERM_BP_FAT | GO:0007049~cell cycle | 282 | 17.59 | 2.56E-31 |
|  | GOTERM_BP_FAT | GO:0022402~cell cycle process | 244 | 15.22 | 3.98E-31 |
|  | GOTERM_BP_FAT | GO:0051276~chromosome organization | 217 | 13.54 | 1.05E-28 |
|  | GOTERM_CC_FAT | GO:0044427~chromosomal part | 183 | 11.42 | 3.40E-33 |
|  | GOTERM_CC_FAT | GO:0005694~chromosome | 197 | 12.29 | 1.27E-32 |
|  | GOTERM_CC_FAT | GO:0070161~anchoring junction | 161 | 10.04 | 2.63E-30 |
|  | GOTERM_CC_FAT | GO:0005912~adherens junction | 157 | 9.79 | 1.72E-29 |
|  | GOTERM_CC_FAT | GO:0031012~extracellular matrix | 128 | 7.99 | 8.88E-27 |
|  | GOTERM_MF_FAT | GO:0044877~macromolecular complex binding | 228 | 14.22 | 1.41E-26 |
|  | GOTERM_MF_FAT | GO:0050839~cell adhesion molecule binding | 114 | 7.11 | 2.20E-25 |
|  | GOTERM_MF_FAT | GO:0032403~protein complex binding | 148 | 9.23 | 2.55E-21 |
|  | GOTERM_MF_FAT | GO:0003677~DNA binding | 342 | 21.33 | 8.47E-19 |
|  | GOTERM_MF_FAT | GO:0003682~chromatin binding | 107 | 6.67 | 1.53E-18 |
| Down-regulated | GOTERM_BP_FAT | GO:0006082~organic acid metabolic process | 236 | 17.28 | 2.03E-88 |
|  | GOTERM_BP_FAT | GO:0043436~oxoacid metabolic process | 216 | 15.81 | 3.16E-80 |
|  | GOTERM_BP_FAT | GO:0019752~carboxylic acid metabolic process | 215 | 15.74 | 5.99E-80 |
|  | GOTERM_BP_FAT | GO:0032787~monocarboxylic acid metabolic process | 166 | 12.15 | 6.94E-70 |
|  | GOTERM_BP_FAT | GO:0055114~oxidation-reduction process | 203 | 14.86 | 8.69E-57 |
|  | GOTERM_CC_FAT | GO:0070062~extracellular exosome | 361 | 26.43 | 1.03E-39 |
|  | GOTERM_CC_FAT | GO:1903561~extracellular vesicle | 362 | 26.50 | 1.25E-39 |
|  | GOTERM_CC_FAT | GO:0043230~extracellular organelle | 362 | 26.50 | 1.35E-39 |
|  | GOTERM_CC_FAT | GO:0072562~blood microparticle | 65 | 4.76 | 9.13E-35 |
|  | GOTERM_CC_FAT | GO:0044421~extracellular region part | 429 | 31.41 | 1.92E-32 |
|  | GOTERM_MF_FAT | GO:0004497~monooxygenase activity | 42 | 3.07 | 4.97E-23 |
|  | GOTERM_MF_FAT | GO:0016614~oxidoreductase activity, acting on CH-OH group of donors | 46 | 3.37 | 3.69E-21 |
|  | GOTERM_MF_FAT | GO:0048037~cofactor binding | 65 | 4.76 | 1.05E-20 |
|  | GOTERM_MF_FAT | GO:0016705~oxidoreductase activity, acting on paired donors, with incorporation or reduction of molecular oxygen | 47 | 3.44 | 2.82E-18 |
|  | GOTERM_MF_FAT | GO:0016616~oxidoreductase activity, acting on the CH-OH group of donors, NAD or NADP as acceptor | 38 | 2.78 | 5.01E-17 |

Table S6.Gene ontology analysis of DEGs between PHHs and hHF-iPSCs

| **Expression** | **Category** | **Term** | **Gene count** | **％** | **P value** |
| --- | --- | --- | --- | --- | --- |
| Up-regulated | GOTERM_BP_FAT | GO:0006082~organic acid metabolic process | 231 | 15.08 | 6.13E-73 |
|  | GOTERM_BP_FAT | GO:0043436~oxoacid metabolic process | 212 | 13.84 | 1.15E-66 |
|  | GOTERM_BP_FAT | GO:0019752~carboxylic acid metabolic process | 211 | 13.77 | 1.94E-66 |
|  | GOTERM_BP_FAT | GO:0032787~monocarboxylic acid metabolic process | 161 | 10.51 | 1.03E-57 |
|  | GOTERM_BP_FAT | GO:0006629~lipid metabolic process | 243 | 15.86 | 1.17E-47 |
|  | GOTERM_CC_FAT | GO:0070062~extracellular exosome | 398 | 25.98 | 1.84E-41 |
|  | GOTERM_CC_FAT | GO:1903561~extracellular vesicle | 399 | 26.04 | 2.54E-41 |
|  | GOTERM_CC_FAT | GO:0043230~extracellular organelle | 399 | 26.04 | 2.77E-41 |
|  | GOTERM_CC_FAT | GO:0072562~blood microparticle | 68 | 4.44 | 1.18E-34 |
|  | GOTERM_CC_FAT | GO:0044421~extracellular region part | 472 | 30.81 | 7.99E-33 |
|  | GOTERM_MF_FAT | GO:0004497~monooxygenase activity | 44 | 2.87 | 7.63E-23 |
|  | GOTERM_MF_FAT | GO:0016705~oxidoreductase activity, acting on paired donors, with incorporation or reduction of molecular oxygen | 51 | 3.33 | 4.67E-19 |
|  | GOTERM_MF_FAT | GO:0005319~lipid transporter activity | 39 | 2.55 | 9.86E-17 |
|  | GOTERM_MF_FAT | GO:0048037~cofactor binding | 63 | 4.11 | 1.35E-16 |
|  | GOTERM_MF_FAT | GO:0016614~oxidoreductase activity, acting on CH-OH group of donors | 43 | 2.81 | 1.55E-16 |
| Down-regulated | GOTERM_BP_FAT | GO:0051276~chromosome organization | 322 | 16.79 | 1.52E-67 |
|  | GOTERM_BP_FAT | GO:0000278~mitotic cell cycle | 265 | 13.82 | 1.96E-55 |
|  | GOTERM_BP_FAT | GO:1903047~mitotic cell cycle process | 247 | 12.88 | 1.15E-52 |
|  | GOTERM_BP_FAT | GO:0022402~cell cycle process | 312 | 16.27 | 1.03E-47 |
|  | GOTERM_BP_FAT | GO:0007049~cell cycle | 355 | 18.51 | 2.85E-46 |
|  | GOTERM_CC_FAT | GO:0005654~nucleoplasm | 602 | 31.39 | 1.65E-74 |
|  | GOTERM_CC_FAT | GO:0044427~chromosomal part | 260 | 13.56 | 2.12E-66 |
|  | GOTERM_CC_FAT | GO:0005694~chromosome | 278 | 14.49 | 3.39E-65 |
|  | GOTERM_CC_FAT | GO:0000228~nuclear chromosome | 175 | 9.12 | 4.45E-44 |
|  | GOTERM_CC_FAT | GO:0044454~nuclear chromosome part | 167 | 8.71 | 2.63E-43 |
|  | GOTERM_MF_FAT | GO:0003676~nucleic acid binding | 673 | 35.09 | 3.32E-49 |
|  | GOTERM_MF_FAT | GO:1901363~heterocyclic compound binding | 878 | 45.7 | 9.76E-48 |
|  | GOTERM_MF_FAT | GO:0097159~organic cyclic compound binding | 886 | 46.19 | 1.57E-47 |
|  | GOTERM_MF_FAT | GO:0003677~DNA binding | 461 | 24.04 | 1.35E-40 |
|  | GOTERM_MF_FAT | GO:0044822~poly(A) RNA binding | 244 | 12.72 | 4.74E-28 |

Table S7. KEGG pathway analysis of DEGs between HLCs and hHF-iPSCs

| **Pathway ID** | **Name** | **Gene count** | **%** | **P Value** | **Genes** |
| --- | --- | --- | --- | --- | --- |
| **Up-regulated DEGs** |  |  |  |  |  |
| hsa04512 | ECM-receptor interaction | 27 | 3.97 | 2.45E-16 | TNC, COL3A1, ITGA11, SDC4, ITGB1, CD47, ITGAV, COL6A3, ITGB6, THBS1, LAMB1, THBS3, FN1, COL4A2, COL4A1, HSPG2, ITGA2, ITGA3, COL5A2, COL5A1, ITGA5, ITGA8, COL1A2, RELN, LAMC2, COL1A1, LAMC1 |
| hsa04510 | Focal adhesion | 38 | 5.59 | 8.50E-15 | TLN2, TNC, COL3A1, ITGA11, ITGB1, ITGAV, SOS2, ITGB6, COL6A3, PDGFC, LAMB1, THBS1, PIK3R1, THBS3, FN1, EGFR, COL4A2, COL4A1, MET, ITGA2, ITGA3, HGF, CAPN2, FLNC, COL5A2, FLNB, COL5A1, ITGA5, ITGA8, COL1A2, PDGFRA, PDGFRB, LAMC2, RELN, COL1A1, LAMC1, MYLK, PARVA |
| hsa05205 | Proteoglycans in cancer | 32 | 4.71 | 8.08E-11 | WNT5A, TNF, LUM, MMP9, DCN, SDC4, MMP2, TIMP3, ITGB1, MIR21, TGFB1, TGFB2, ITGAV, SOS2, CAMK2D, RRAS, THBS1, PIK3R1, FN1, EGFR, MET, HSPG2, ITGA2, HGF, FLNC, ITPR3, FLNB, PLAUR, FZD6, ITGA5, HBEGF, PLAU |
| hsa04151 | PI3K-Akt signaling pathway | 38 | 5.59 | 4.47E-08 | OSMR, EFNA1, TNC, COL3A1, ITGA11, ITGB1, ITGAV, SOS2, ITGB6, COL6A3, CREB3L2, CREB3L1, PDGFC, LAMB1, THBS1, PIK3R1, THBS3, FN1, EGFR, COL4A2, COL4A1, MET, ITGA2, NR4A1, ITGA3, HGF, COL5A2, COL5A1, ITGA5, ITGA8, COL1A2, PDGFRA, JAK1, PDGFRB, LAMC2, RELN, COL1A1, LAMC1 |
| hsa05412 | Arrhythmogenic right ventricular cardiomyopathy (ARVC) | 14 | 2.06 | 4.57E-06 | ITGA11, ITGA2, LEF1, CACNG4, ITGA3, ITGB1, JUP, PKP2, ITGA5, ITGAV, ITGA8, ITGB6, DSC2, DSP |
| **Down-regulated DEGs** |  |  |  |  |  |
| hsa05322 | Systemic lupus erythematosus | 42 | 5.61 | 6.74E-24 | HIST1H2AB, HIST1H4L, HIST4H4, HIST1H4K, HIST1H2AG, HIST1H2AE, SNRPD1, HIST2H4A, HIST2H4B, HIST1H2BO, HIST2H2AB, HIST1H2BM, HIST1H2BK, HIST1H4A, HIST1H4B, HIST1H2BI, HIST1H2BJ, H2AFZ, HIST1H4E, HIST1H4F, HIST1H4C, HIST1H4D, HIST1H4I, HIST1H4J, HIST1H4H, HIST2H3A, HIST1H3J, HIST1H2BE, HIST1H2BF, HIST1H2BG, HIST2H3D, HIST1H3A, HIST1H3B, HIST1H2AI, HIST1H3C, HIST1H3D, HIST1H3E, HIST1H3F, HIST1H2AM, HIST1H3G, HIST1H3H, HIST1H3I |
| hsa05034 | Alcoholism | 42 | 5.61 | 6.40E-19 | HIST1H2AB, HIST1H4L, HIST4H4, HIST1H4K, HIST1H2AG, HIST1H2AE, HIST2H4A, HIST2H4B, HIST1H2BO, HIST2H2AB, HIST1H2BM, HIST1H2BK, HIST1H4A, HIST1H4B, HIST1H2BI, HIST1H2BJ, H2AFZ, HIST1H4E, HIST1H4F, HIST1H4C, HIST1H4D, HIST1H4I, HIST1H4J, HIST1H4H, HIST2H3A, HIST1H3J, HIST1H2BE, HIST1H2BF, HIST1H2BG, PPP1CC, HIST2H3D, HIST1H3A, HIST1H3B, HIST1H2AI, HIST1H3C, HIST1H3D, HIST1H3E, HIST1H3F, HIST1H2AM, HIST1H3G, HIST1H3H, HIST1H3I |
| hsa05203 | Viral carcinogenesis | 32 | 4.27 | 2.11E-09 | USP7, HIST1H4L, HIST4H4, HIST1H4K, CHEK1, PMAIP1, HIST2H4A, HIST2H4B, HIST1H2BO, CCNE2, CASP3, HIST1H2BM, HIST1H2BK, HIST1H4A, HIST1H4B, HIST1H2BI, HIST1H2BJ, HIST1H4E, HIST1H4F, HIST1H4C, HIST1H4D, HIST1H4I, CCNA2, HIST1H4J, HIST1H4H, HIST1H2BE, HIST1H2BF, HIST1H2BG, TP53, CDK4, CCND1, RBPJ |
| hsa04110 | Cell cycle | 23 | 3.07 | 2.71E-08 | CDC7, ANAPC1, CDC6, DBF4, TP53, TTK, CHEK1, MCM2, CDK4, MCM3, MCM4, CDC25A, MCM5, MCM6, CCNE2, CCND1, MAD2L1, MCM7, BUB1, PCNA, ORC1, CCNA2, ORC3 |
| hsa03030 | DNA replication | 13 | 1.74 | 2.84E-08 | POLA1, MCM2, RNASEH2A, MCM3, MCM4, MCM5, MCM6, PRIM1, RFC3, MCM7, RFC2, PRIM2, PCNA |

Table S8. KEGG pathway analysis of DEGs between HLCs and PHHs

| **Pathway ID** | **Name** | **Gene count** | **%** | **P Value** | **Genes** |
| --- | --- | --- | --- | --- | --- |
| **Up-regulated DEGs** |  |  |  |  |  |
| hsa04110 | Cell cycle | 45 | 2.81 | 6.27E-16 | E2F3, YWHAZ, DBF4, PRKDC, TTK, CHEK1, SFN, TGFB1, TGFB2, CCNE1, MCM7, BUB1, ORC6, ORC1, CCNA2, MYC, ORC3, CDC7, CDK1, CDC6, SKP2, CDC23, SMAD3, CDC20, SMAD2, MCM3, CDC25C, MCM4, MCM5, CDC25A, CDK2, WEE1, MCM6, CCNB1, HDAC2, CCNB2, MAD2L1, PLK1, CCND2, GSK3B, PCNA, YWHAQ, MDM2, ABL1, GADD45A |
| hsa05322 | Systemic lupus erythematosus | 45 | 2.81 | 1.62E-14 | HIST1H2AB, HIST1H4L, HIST2H2AA4, HIST4H4, TNF, HIST1H4K, HIST1H2AE, HIST2H4A, HIST2H4B, HIST1H2BO, HIST2H2AB, HIST1H2BM, HIST1H2BK, HIST1H4A, HIST1H4B, HIST2H2AC, HIST1H2BI, HIST1H2BJ, HIST1H4E, HIST1H4F, H2AFX, HIST1H4C, HIST1H4D, HIST1H4I, HIST1H4J, HIST1H4H, HIST2H3A, HIST1H3J, ACTN4, HIST1H2BF, HIST1H2BH, ACTN1, HIST2H3D, HIST1H3A, H2AFY2, HIST1H3B, HIST1H3C, H3F3B, HIST1H3D, HIST1H3E, HIST1H3F, HIST1H2AM, HIST1H3G, HIST1H3H, HIST1H3I |
| hsa05034 | Alcoholism | 49 | 3.06 | 3.25E-12 | HIST1H2AB, HIST1H4L, HIST4H4, HIST2H2AA4, HIST1H4K, HIST1H2AE, HIST2H4A, HIST2H4B, HIST1H2BO, HIST2H2AB, HIST1H2BM, HIST1H4A, HIST1H2BK, HIST1H4B, HIST1H2BI, HIST2H2AC, HIST1H2BJ, HIST1H4E, CREB3L2, CREB3L1, HIST1H4F, H2AFX, HIST1H4C, HIST1H4D, HIST1H4I, HIST1H4J, HIST1H4H, HIST2H3A, HIST1H3J, HIST1H2BF, HIST1H2BH, FOSB, HIST2H3D, NRAS, HDAC2, H2AFY2, HIST1H3A, HIST1H3B, HIST1H3C, CALM3, GNB4, H3F3B, HIST1H3D, HIST1H3E, HIST1H3F, HIST1H2AM, HIST1H3G, HIST1H3H, HIST1H3I |
| hsa05205 | Proteoglycans in cancer | 52 | 3.24 | 8.55E-12 | MMP9, MMP2, TGFB1, IQGAP1, CTNNB1, TGFB2, ACTG1, GPC3, TIAM1, RRAS, PAK1, MYC, FRS2, AKT3, FLNC, FLNB, FLNA, PLAUR, HIF1A, RRAS2, MDM2, WNT5A, FGFR1, TNF, LUM, ERBB2, MIR21, TIMP3, PXN, DROSHA, IGF1R, PTK2, EZR, ITGAV, PPP1R12A, THBS1, FN1, ACTB, CBL, HSPG2, ITGA2, FZD3, ITPR3, FZD7, FZD6, NRAS, PLCG1, ITGA5, HBEGF, PTCH1, PLAU, SLC9A1 |
| hsa04510 | Focal adhesion | 52 | 3.24 | 2.82E-11 | TLN2, VCL, CTNNB1, PAK6, ACTG1, ILK, PAK1, AKT3, ACTN4, ACTN1, FLNC, FLNB, VASP, FLNA, CCND2, JUN, COL1A2, LAMC2, LAMC1, COL1A1, PARVA, TNC, ERBB2, COL3A1, ITGA11, PXN, MYL9, IGF1R, PTK2, DOCK1, ITGAV, ITGB6, COL6A3, PPP1R12A, LAMB1, THBS1, FN1, SPP1, ACTB, COL4A2, COL4A1, ITGA2, ITGA3, CAPN2, COL5A2, COL5A1, COL4A5, LAMA1, ITGA6, ITGA5, ITGA8, GSK3B |
| **Down-regulated DEGs** |  |  |  |  |  |
| hsa04610 | Complement and coagulation cascades | 50 | 3.66 | 2.64E-37 | C7, C3AR1, MBL2, A2M, C9, MASP1, C3, MASP2, C6, C5, C1R, C1S, C1QC, F13B, FGG, FGA, FGB, SERPINA5, KLKB1, SERPINC1, CFH, SERPINA1, C2, CFI, F11, KNG1, F12, CR1, F10, C4B, CFB, F8, F9, C4BPB, SERPING1, F7, C4BPA, PLG, C8G, PROC, C8A, C8B, C1QB, F5, SERPINF2, F2, TFPI, SERPIND1, CPB2, PROS1 |
| hsa01100 | Metabolic pathways | 215 | 15.74 | 6.80E-29 | CYP3A4, CYP3A5, LDHA, GDA, CYP3A7, EHHADH, GLDC, FAH, MAT1A, CYP7A1, ACSM2B, ACAA2, GATM, SUCLG2, ACSM2A, CYP1A2, CYP2E1, CD38, GLUL, BHMT, PGM1, PLA2G2A, HSD11B1, ABAT, ACADSB, ENPP1, CYP2B6, ALDOB, ACAT1, ARG1, IDH2, CDA, ALDH4A1, FH, ST6GAL1, UAP1, CYP2C9, CYP2C8, MAOA, UPB1, MAOB, HGD, ACACB, CYP17A1, GBE1, HMGCS2, PON1, PON2, AGXT2, GK, CYP8B1, PON3, NNMT, SEPHS2, CYP2J2, CYP2C19, CYP2C18, ANPEP, MTHFD1, NT5E, HYAL1, MINPP1, PIK3C2G, ACO1, GRHPR, HAO1, G6PC, CYP27A1, SDS, H6PD, ADK, HAO2, TGDS, SLC27A5, ALPL, SORD, HSD17B2, HSD3B7, UGDH, DPYS, GCH1, ACSL1, PLA2G12B, HSD17B6, HSD17B4, PAPSS2, HSD17B7, AGL, ACSL5, SHMT1, CES1, NAT2, PCK1, GBA3, KHK, ACSM3, SDHB, GANC, AOX1, SDHD, GAMT, DPYD, ACSM5, CNDP1, ADH1C, ADH1B, ADH1A, ACSS3, AGXT, ST3GAL6, RGN, SPR, DAO, ALDH6A1, SPTLC3, SLC33A1, FBP1, HAL, CDO1, PNPLA3, CHPT1, TAT, PCCA, XDH, GCLC, ASS1, GLUD2, GNE, GLUD1, PAH, KMO, AGMAT, CMPK1, GALM, MUT, CERS2, DHODH, UGT2B28, ACY1, GALT, EPHX2, ACMSD, AK2, AK4, CPS1, IDH3A, BAAT, NDUFV2, ALDH2, DCXR, PC, ACOX2, NAMPT, ACOX1, KYNU, SAT2, ALG5, AFMID, AKR1C3, CRYL1, TDO2, ALAS1, AKR1C4, MGLL, HPD, DDC, ACADM, ALDH5A1, OTC, MAN1A1, LPIN2, ACADL, LPIN1, LAP3, ACADVL, MAN2A2, FOLH1, DHRS3, UGT2B17, DGAT2, PKLR, UGT2B11, CYP2A6, UGT2B10, UGT2B15, AKR1D1, GPAM, XYLB, ADH6, ECHS1, COMT, ALDH3A2, PIPOX, ALDH1A1, MTHFS, UGT1A5, ADH4, PNPO, DMGDH, UGT2A3, BDH1, CYP21A2, ADI1, CYP4A11, AMDHD1, GPI, LIPG, UGT2B4, CYP4F3, HIBCH, CYP4F2, RDH16, LIPC, SCP2, UGT2B7 |
| hsa00982 | Drug metabolism - cytochrome P450 | 35 | 2.56 | 4.46E-19 | CYP3A4, CYP3A5, CYP2C19, CYP2B6, CYP2D6, ADH1C, ADH1B, ADH6, ADH1A, FMO4, FMO5, ADH4, FMO2, UGT1A5, FMO3, UGT2A3, UGT2B28, GSTA1, GSTA2, CYP2C9, CYP2C8, MAOA, MAOB, CYP2E1, CYP1A2, UGT2B17, AOX1, UGT2B11, UGT2B4, CYP2A6, UGT2B10, UGT2B15, MGST1, UGT2B7, MGST2 |
| hsa00980 | Metabolism of xenobiotics by cytochrome P450 | 34 | 2.49 | 1.19E-16 | CYP3A4, CYP3A5, CYP2B6, CYP2D6, ADH1C, ADH1B, ADH6, ADH1A, CYP2A13, AKR1C2, AKR1C4, ADH4, UGT1A5, AKR7A3, UGT2A3, UGT2B28, AKR1C1, GSTA1, GSTA2, SULT2A1, CYP2C9, EPHX1, CYP2E1, CYP1A2, UGT2B17, HSD11B1, UGT2B11, UGT2B4, CYP2A6, UGT2B10, UGT2B15, MGST1, UGT2B7, MGST2 |
| hsa00140 | Steroid hormone biosynthesis | 30 | 2.20 | 1.88E-16 | CYP3A4, CYP3A5, CYP3A7, HSD17B2, COMT, AKR1C3, AKR1C2, AKR1C4, UGT1A5, CYP7A1, HSD17B6, SRD5A2, SULT1E1, UGT2A3, UGT2B28, HSD17B7, AKR1C1, CYP21A2, CYP2E1, CYP1A2, CYP7B1, UGT2B17, CYP17A1, HSD11B1, UGT2B11, UGT2B4, UGT2B10, UGT2B15, AKR1D1, UGT2B7 |

Table S9. KEGG pathway analysis of DEGs between PHHs and hHF-iPSCs

| **Pathway ID** | **Name** | **Gene count** | **%** | **P Value** | **Genes** |
| --- | --- | --- | --- | --- | --- |
| **Up-regulated DEGs** |  |  |  |  |  |
| hsa04610 | Complement and coagulation cascades | 50 | 3.26 | 2.67E-35 | C7, C3AR1, MBL2, A2M, C9, MASP1, C3, MASP2, C6, C5, C1R, C1S, C1QC, F13B, FGG, FGA, FGB, SERPINA5, KLKB1, SERPINC1, CFH, SERPINA1, C2, CFI, F11, KNG1, F12, CR1, F10, C4B, CFB, F8, F9, C4BPB, SERPING1, F7, C4BPA, PLG, C8G, PROC, C8A, C8B, C1QB, F5, SERPINF2, F2, TFPI, SERPIND1, CPB2, PROS1 |
| hsa01100 | Metabolic pathways | 211 | 13.77 | 3.98E-21 | CYP3A4, CYP3A5, ALAD, LDHA, GDA, CYP3A7, EHHADH, NMRK1, FAH, MAT1A, CYP7A1, ACSM2B, ACAA2, GATM, SUCLG2, ACSM2A, CYP1A2, CYP2E1, CD38, GLUL, NNT, BHMT, PLA2G2A, HSD11B1, ABAT, EXT1, ACAA1, ACADSB, CYP2B6, ALDOB, ACAT1, ARG1, IDH2, ALDH4A1, ST6GAL1, UAP1, MOCS2, CYP2C9, CYP2C8, MAOA, UPB1, MAOB, HGD, ACACB, CYP17A1, GBE1, HMGCS2, PON1, PON2, AGXT2, GK, CYP8B1, PON3, NNMT, CYP2J2, CYP2C19, CYP2C18, ANPEP, NT5E, AMY2A, HYAL1, MINPP1, PIK3C2G, ACO1, GRHPR, HAO1, G6PC, CYP27A1, SDS, H6PD, ADK, HAO2, SLC27A5, HSD17B2, HSD3B7, UGDH, DPYS, GCH1, ACSL1, PLA2G12B, HSD17B6, PAPSS2, MTMR4, HSD17B7, AGL, ACSL5, SHMT1, CES1, NAT2, PCK1, GBA3, KHK, ACSM3, SDHB, GANC, AOX1, GAMT, DPYD, ACSM5, IMPAD1, CNDP1, ADH1C, ADH1B, ADH1A, ACSS3, AGXT, ST3GAL1, ST3GAL6, RGN, SPR, DAO, ALDH6A1, SPTLC3, SLC33A1, FBP1, HAL, CDO1, PNPLA3, CHPT1, TAT, GNS, PCCA, XDH, ASS1, GLUD2, GNE, GLUD1, PAH, KMO, AGMAT, CMPK1, GALM, MUT, GMPPA, DHODH, UGT2B28, B4GALT1, ACY1, MSMO1, GALT, EPHX2, ACMSD, CPS1, BAAT, ALDH2, DCXR, PC, ACOX2, SAT1, ACOX1, NAMPT, KYNU, SAT2, ALG5, AFMID, AKR1C3, CRYL1, TDO2, AKR1C4, MGLL, HPD, DDC, MAN1A2, ALDH5A1, OTC, MAN1A1, LPIN2, ACADL, LPIN1, ACADVL, MAN2A2, DHRS3, UGT2B17, FOLH1, DGAT2, PKLR, UGT2B11, CYP2A6, UGT2B10, UGT2B15, GPAM, AKR1D1, XYLB, GALNT2, ADH6, ECHS1, COMT, PIPOX, ALDH1A1, UGT1A5, ADH4, DAD1, PNPO, DMGDH, UGT2A3, BDH1, CYP21A2, ADI1, CYP4A11, AMDHD1, RDH10, SMPD1, LIPG, UGT2B4, CYP4F3, HIBCH, CYP4F2, RDH16, LIPC, SCP2, UGT2B7 |
| hsa00982 | Drug metabolism - cytochrome P450 | 35 | 2.28 | 8.72E-18 | CYP3A4, CYP3A5, CYP2C19, CYP2B6, CYP2D6, ADH1C, ADH1B, ADH6, ADH1A, FMO4, FMO5, ADH4, FMO2, UGT1A5, GSTK1, FMO3, UGT2A3, UGT2B28, GSTA1, GSTA2, CYP2C9, CYP2C8, MAOA, MAOB, CYP2E1, CYP1A2, UGT2B17, AOX1, UGT2B11, UGT2B4, CYP2A6, UGT2B10, UGT2B15, UGT2B7, MGST2 |
| hsa00980 | Metabolism of xenobiotics by cytochrome P450 | 34 | 2.22 | 2.19E-15 | CYP3A4, CYP3A5, CYP2B6, CYP2D6, ADH1C, ADH1B, ADH6, ADH1A, CYP2A13, AKR1C2, AKR1C4, ADH4, UGT1A5, GSTK1, AKR7A3, UGT2A3, UGT2B28, AKR1C1, GSTA1, GSTA2, SULT2A1, CYP2C9, EPHX1, CYP2E1, CYP1A2, UGT2B17, HSD11B1, UGT2B11, UGT2B4, CYP2A6, UGT2B10, UGT2B15, UGT2B7, MGST2 |
| hsa00140 | Steroid hormone biosynthesis | 30 | 1.96 | 2.57E-15 | CYP3A4, CYP3A5, CYP3A7, HSD17B2, COMT, AKR1C3, AKR1C2, AKR1C4, UGT1A5, CYP7A1, HSD17B6, SRD5A2, SULT1E1, UGT2A3, UGT2B28, HSD17B7, AKR1C1, CYP21A2, CYP2E1, CYP1A2, CYP7B1, UGT2B17, CYP17A1, HSD11B1, UGT2B11, UGT2B4, UGT2B10, UGT2B15, AKR1D1, UGT2B7 |
| **Down-regulated DEGs** |  |  |  |  |  |
| hsa04110 | Cell cycle | 57 | 2.97 | 1.91E-22 | E2F3, E2F4, E2F5, DBF4, TTK, PTTG1, CDC16, CCNE2, CCNE1, CDC45, MCM7, RAD21, ORC6, MYC, ORC1, CCNA2, STAG2, ORC2, ORC3, CDC7, ANAPC1, CDK1, CDC6, RBL1, TP53, SKP2, MCM2, CDK7, CDK4, MCM3, MCM4, MCM5, WEE1, MCM6, CCND1, MAD2L1, CCND2, MAD2L2, YWHAZ, PRKDC, CHEK1, BUB1, BUB3, CDC23, SMAD2, CDC20, CDC25C, CDC25A, SMC3, CCNB1, YWHAG, HDAC2, CCNB2, PLK1, PCNA, YWHAQ, ABL1 |
| hsa05322 | Systemic lupus erythematosus | 55 | 2.87 | 6.60E-19 | HIST2H2AA4, HIST4H4, SNRPD1, HIST2H4A, HIST2H4B, HIST1H2BO, HIST2H2AB, HIST1H2BM, HIST1H2BK, HIST1H2BL, HIST2H2AC, HIST1H2BI, HIST1H2BJ, H2AFZ, HIST3H2A, H2AFX, SNRPB, HIST1H2AB, HIST1H4L, HIST1H4K, HIST1H2AG, HIST1H2AE, HIST1H4A, HIST1H4B, HIST1H4E, HIST1H4F, HIST1H4C, HIST1H4D, HIST1H4I, HIST1H4J, HIST1H4H, HIST2H3A, HIST1H3J, HIST1H2BE, HIST1H2BF, HIST1H2BG, HIST1H2BH, SSB, HIST2H3D, HIST1H3A, H2AFY2, HIST1H3B, HIST1H2AI, HIST1H2AH, H3F3A, HIST1H3C, HIST1H3D, HIST1H2AK, H3F3B, HIST1H3E, HIST1H3F, HIST1H2AM, HIST1H3G, HIST1H3H, HIST1H3I |
| hsa05034 | Alcoholism | 58 | 3.02 | 1.29E-14 | HIST2H2AA4, HIST4H4, HIST2H4A, HIST2H4B, HIST1H2BO, HIST2H2AB, HIST1H2BM, HIST1H2BK, HIST1H2BL, HIST2H2AC, HIST1H2BI, HIST1H2BJ, H2AFZ, HIST3H2A, H2AFX, GNG4, PPP1CC, GNB4, HIST1H2AB, HIST1H4L, HIST1H4K, HIST1H2AG, HIST1H2AE, HIST1H4A, HIST1H4B, HIST1H4E, HIST1H4F, HIST1H4C, HIST1H4D, HIST1H4I, HIST1H4J, HIST1H4H, HIST2H3A, HIST1H3J, HIST1H2BE, HIST1H2BF, HIST1H2BG, HIST1H2BH, HIST2H3D, NRAS, HDAC2, HIST1H3A, H2AFY2, HIST1H3B, HIST1H2AI, CALM3, HIST1H2AH, H3F3A, HIST1H3C, HIST1H3D, HIST1H2AK, H3F3B, HIST1H3E, HIST1H3F, HIST1H2AM, HIST1H3G, HIST1H3H, HIST1H3I |
| hsa03030 | DNA replication | 21 | 1.09 | 7.33E-11 | POLA1, POLA2, MCM2, RNASEH2A, MCM3, MCM4, MCM5, MCM6, RFC5, POLD3, RPA1, PRIM1, DNA2, RFC3, RFC4, MCM7, POLE2, RFC2, PRIM2, PCNA, FEN1 |
| hsa05203 | Viral carcinogenesis | 51 | 2.66 | 3.05E-08 | HIST4H4, PMAIP1, HIST2H4A, HIST2H4B, CCNE2, PKM, HIST1H2BO, GTF2E1, CCNE1, HIST1H2BM, CASP3, HIST1H2BK, HIST1H2BL, HIST1H2BI, HIST1H2BJ, CCNA2, CDK1, RBL1, TP53, SKP2, CDK4, CCND1, CCND2, JUN, USP7, YWHAZ, HIST1H4L, HIST1H4K, CHEK1, PXN, HIST1H4A, HIST1H4B, HIST1H4E, HIST1H4F, HIST1H4C, HIST1H4D, HIST1H4I, HIST1H4J, TRAF5, CHD4, HIST1H4H, HIST1H2BE, HIST1H2BF, HIST1H2BG, HIST1H2BH, CDC20, NRAS, YWHAG, HDAC2, YWHAQ, RBPJ |
