# Supplementary figures and images for: Analysis of differentially expressed genes among human hair follicle–derived iPSCs, induced hepatocyte-like cells, and primary hepatocytes
Source: Stem Cell Res Ther. 2018 Aug 9;9:211. doi: 10.1186/s13287-018-0940-z (PMC6085644; doi:10.1186/s13287-018-0940-z)

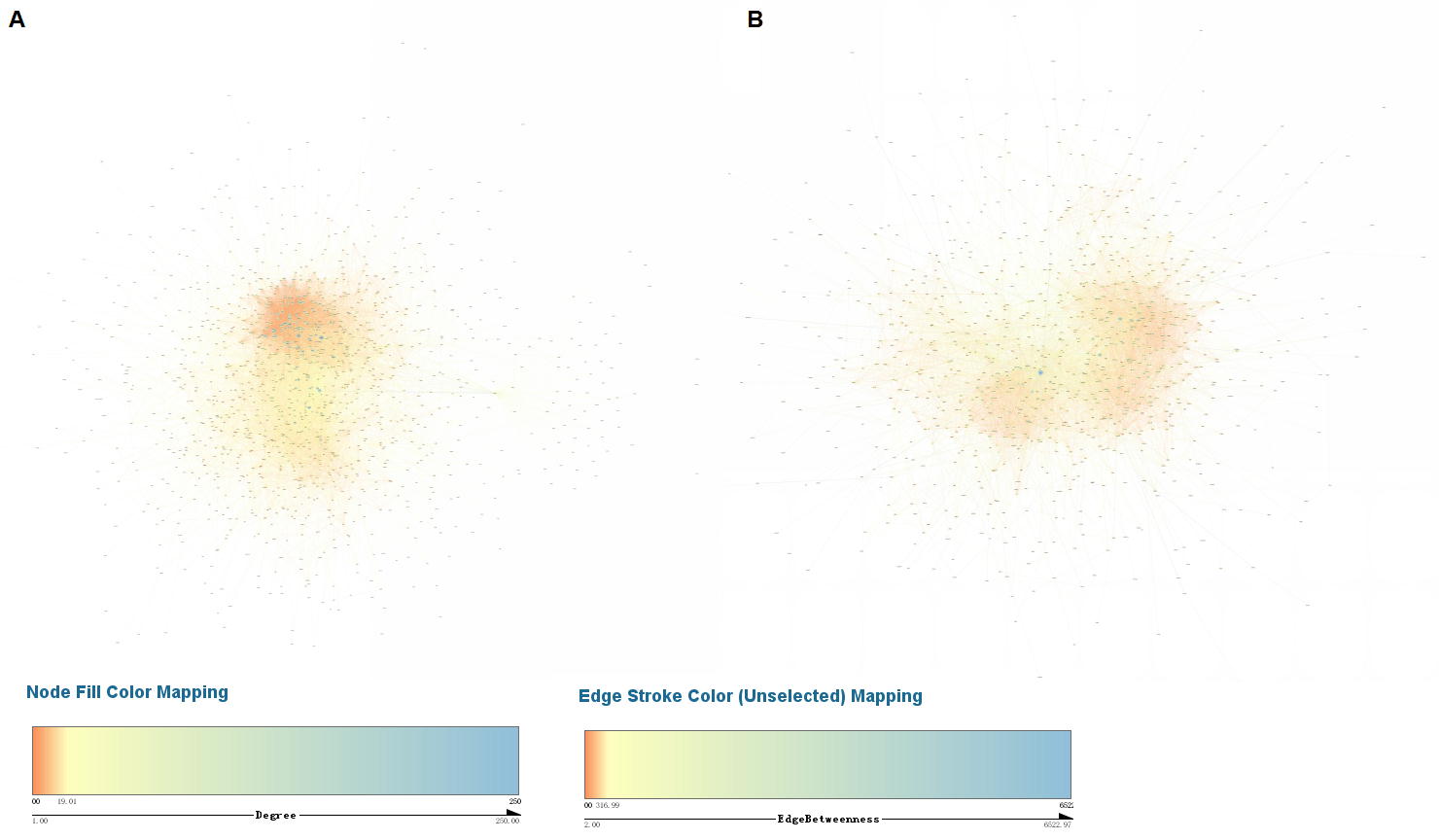

Supplement: Supplementary file 2 — PPI network and module analysis in HLCs/PHHs. (A) Network of all upregulated DEGs. (B) Network of all downregulated DEGs. Different colors, node size, and edge width show the node’s degree and the edge’s edge-betweenness. (JPG 64 kb) [file 13287_2018_940_MOESM2_ESM.jpg]
